# Impact of population aging on the burden of vaccine-preventable diseases among older adults in the United States

**DOI:** 10.1080/21645515.2020.1780847

**Published:** 2020-08-06

**Authors:** Sandra E Talbird, Elizabeth M La, Justin Carrico, Sara Poston, Jean-Etienne Poirrier, Jessica K DeMartino, Cosmina S Hogea

**Affiliations:** a Health Economics, RTI Health Solutions, Research Triangle Park, NC, USA; b US Health Outcomes & Epidemiology, Vaccines, GSK, Philadelphia, PA, USA; c Global Value Evidence and Outcomes, Oncology,GSK, Philadelphia, PA, USA

**Keywords:** Infectious diseases, economic burden, vaccines costs and spending, aging populations

## Abstract

Despite vaccination recommendations, the burden of vaccine-preventable diseases remains high in older adults in the United States (US), contributing to substantial morbidity, mortality, and health care resource use and costs. To adequately plan for health care resource needs and to help inform vaccination policies, burden of disease projections that account for population aging over the coming decades are needed. As a first step, this exploratory study projects the burden of influenza, pertussis, herpes zoster, and pneumococcal disease in adults aged 50 y and older in the US, using a population-based modeling framework with separate decision trees for each vaccine-preventable disease. The model uses projected population estimates from the US Census Bureau to account for changes in the US population over time and then calculates expected numbers of cases and associated costs for each disease, keeping current estimates of age-specific disease incidence, vaccine coverage, and efficacy constant over time. This approach was used to focus the exploratory analysis on the burden of disease that may be expected due to population changes alone, assuming that all else remains unchanged. Due to population growth and the shifting age distribution over the next 30 y, the annual societal economic burden for the four vaccine-preventable diseases is projected to increase from approximately $35 billion to $49 billion, resulting in cumulative costs of approximately $1.3 trillion, as well as more than 1 million disease-related deaths. Given such notable burden, further efforts to increase vaccination coverage and effectiveness in older adults are needed.

## Introduction

The human and economic burden of vaccine-preventable diseases in the United States (US) remains high among adults,^1,2^ despite vaccination recommendations from the Centers for Disease Control and Prevention (CDC) and the Advisory Committee on Immunization Practices (ACIP).^3^ In 2016, national estimates of vaccination coverage for four key adult vaccines (influenza; tetanus, diphtheria, and acellular pertussis [Tdap]; herpes zoster [HZ]; and pneumococcal disease) were generally low, ranging from 20.4% for Tdap vaccine to 70.4% for influenza vaccine among individuals aged 65 y and older.^4^ As a result, vaccine-preventable diseases continue to be associated with substantial morbidity, mortality, health care resource use, and costs nationally.^1,2^

Two previously published models have estimated the human and economic burden of these four vaccine-preventable diseases among US adults aged 50 y and older, with annual societal perspective cost estimates ranging from approximately 6.9 USD billion to 26.5 USD billion.^1,2^ However, in addition to producing substantially different burden of disease estimates, these models did not project estimates beyond a period of 1 y. To adequately plan for future health care needs and to help inform vaccination policies, long-range projections of disease burden need to account for population growth and shifts in the US population age distribution (i.e., growth in the percentage of the population over the age of 50 y; referred to as “population aging” throughout). Although previous efforts have projected the burden of disease for HZ^5^ and pneumococcal disease^6^ separately, a consistent modeling framework has not yet been used to project the burden across diseases.

The objective of this exploratory study was to use a consistent population-based modeling framework to project the clinical and economic burden of four vaccine-preventable diseases in US adults aged 50 y and older based on demographic changes alone (i.e., holding all else constant).

## Patients and methods

### Model overview

A population-based burden of disease model that accounts for the impact of population aging was built in Excel to explore the projected burden of influenza, pertussis, HZ, and pneumococcal disease in the US over a time horizon of up to 30 y. Consistent with the model by Ozawa et al.,^2^ the underlying model structure consists of separate decision trees for each vaccine-preventable disease, accounting for disease severity and resource use. The decision tree model structures can be found in Supplementary Figures S1-S4.

For each disease, the population is divided into an unvaccinated and a vaccinated population based on national estimates for vaccine coverage in 2016. Unvaccinated individuals are at risk for each disease, and vaccinated individuals are at reduced risk for each disease using corresponding vaccine efficacy and waning estimates (waning not included for the annual influenza vaccine). Case-severity distributions are included for each disease to appropriately capture costs of cases by severity. Individuals who develop cases of influenza, pertussis, and pneumococcal disease are at risk of disease-related death. Death from HZ is assumed negligible and is not modeled. The model assumes that vaccination reduces the probability of developing a case of disease, but does not affect the severity of disease if a case develops, despite evidence that vaccinated individuals may experience milder forms of disease as compared with unvaccinated individuals.^7–10^

The model uses projected population estimates by 1-y age groups taken directly from the US Census Bureau^11^ to account for projected changes in the US population over 30 y (2017–2046). The model applies current estimates of disease incidence, disease-related mortality, vaccination coverage, vaccine efficacy, and disease-related costs (all held constant over the modeled time horizon) to isolate the impact of population aging on projected disease burden. Disease incidence was derived for unvaccinated individuals from surveillance data or taken from the published literature. Reported pertussis incidence from surveillance data was adjusted to account for underreporting by a factor of 100;^12–16^ this was tested in sensitivity analysis ranging the underreporting factor from 1 (no underreporting) to 200. Where possible, the model uses pneumococcal disease incidence data from 2010 to 2013 (following recommended use of 13-valent pneumococcal conjugate vaccine [PCV-13] in infants starting in 2010 and prior to recommended use of PCV-13 in adults starting in 2014) to isolate the impact of adult vaccination with PCV-13. A summary of disease-specific and vaccine-specific input parameters and ranges used in sensitivity analyses can be found in Table 1. A complete listing of disease-specific input parameters (incidence, case-severity distributions, percentages of cases resulting in death, direct medical costs per case); vaccine-specific input parameters (coverage, efficacy, waning); and societal perspective cost input parameters (over-the-counter medication costs, productivity losses due to mortality or cases of disease) can be found in Supplementary Tables S1-S7.

**Table 1.** Key input parameters

Input Parameter	Default Values (Ranges Used in Sensitivity Analyses)^a^				References
	Influenza	Pertussis	Pneumococcal^b^	HZ	
Vaccination coverage	% Vaccinated each year	% Vaccinated in last 10 years	% Ever vaccinated since 2014	% Ever vaccinated since 2006	^17,18^
Ages 50–59 y	44.8%	28.0%	N/A	0.0%^c^	
	(42.7–47.0%)	(26.6–29.5%)			
Ages 60–64 y	44.8%	28.0%	N/A	23.9%	
	(42.7–47.0%)	(26.6–29.5%)		(21.7–26.2%)	
Ages 65+ y	65.4%	20.4%	66.9%	37.4%	
	(63.4–66.7%)	(18.7–22.3%)	(33.5–68.5%)	(35.8–38.9%)	
Incidence per 100,000					^12,14,19-29^
Ages 50–59 y	8,218	250.3	IPD: 17.4 (16.3–18.5)	674	
	(1,385–17,252)	(2.5–500.6)	NBPP: 257.5 (250.8–264.1)	(666–682)	
Ages 60–64 y	8,218	250.3	IPD: 17.4 (16.3–18.5)	932	
	(1,385–17,252)	(2.5–500.6)	NBPP: 257.5 (250.8–264.1)	(920–944)	
Ages 65–69 y	9,264	174.1	IPD: 34.5 (32.7–36.6)	932	
	(3,198–19,406)	(1.7–348.2)	NBPP: 1,015.6 (992.1–1,039.0)	(920–944)	
Ages 70–79 y	9,264	174.1	IPD: 34.5 (32.7–36.6)	1,202	
	(3,198–19,406)	(1.7–348.2)	NBPP: 1,015.6 (992.1–1,039.0)	(1,179–1,225)	
Ages 80+ y	9,264	174.1	IPD: 34.5 (32.7–36.6)	1,278	
	(3,198–19,406)	(1.7–348.2)	NBPP: 1,015.6 (992.1–1,039.0)	(1,249–1,307)	
Vaccine efficacy	43.9%	70.0%	PCV-13:	ZVL:	^12,30-36^
	(19.0-60.0%)	(61.0-82.0%)	IPD: 51.8%	Age 50–69 y: 63.9%	
			(22.4-70.7%)	(56.0-71.0%)	
			NBPP: 24.1%	Age 70+ y: 30.0%	
			(0.0-45.8%)	(20.0-50.0%)	
			PPSV-23:	RZV:	
			IPD: 74%	Age 50–69 y: 95.8%	
			(55-86%)	(83.8-99.7%)	
			NBPP: 54%	Age 70+ y: 89.1%	
			(16-75%)	(72.6-96.6%)	
Duration of protection^d^	N/A	5 y	PCV-13: 15 y	ZVL:	^27,32,33,35-39^
		(3–7 y)	(10–20 y)	Age 50–69 y: 12 y	
			PPSV-23: 5 y	(11–16 y)	
			(0 y NBPP only) (15 y, IPD & NBPP)	Age 70+ y: 6 y	
				(5–8 y)	
				RZV:	
				Age 50–69 y: 30 y	
				(19–60 y)	
				Age 70+ y: 22 y	
				(14–43 y)	

HZ, herpes zoster; IPD, invasive pneumococcal disease; N/A, not applicable; NBPP, nonbacteremic pneumococcal pneumonia; PCV-13, pneumococcal conjugate vaccine; PPSV-23, pneumococcal polysaccharide vaccine; RZV, recombinant zoster vaccine; ZVL, zoster vaccine live.

Note: Additional details on how point estimates were derived from the original source are provided in Supplementary Tables S1-S4.

^a^Ranges are 95% confidence intervals except for the following parameters where clinically plausible ranges were used: all influenza parameters used a low and high point estimate across influenza seasons for the lower and upper bound ranges to reflect the seasonality of the disease and to capture a broader range of uncertainty; for pertussis incidence, a clinically plausible underreporting factor was used ranging from 1 (no underreporting) to 200; for pneumococcal vaccine coverage, a lower bound assumption was made that half of 66.9% of people who reported “ever had a pneumonia shot” received both PCV-13 and PPSV-23; for all duration of protection and waning parameters, clinically plausible ranges across studies or based on assumptions in other economic models were used.

^b^For the first 5 y, the model uses the higher efficacy of either PCV-13 or PPSV-23 (because individuals are assumed to receive both vaccines) with efficacy waning linearly and PPSV-23 waned completely after 5 y. From Years 5 to 15, efficacy for PCV-13 (alone) wanes linearly.

^c^Assumed that HZ vaccine coverage for people ages 50–59 y reaches current coverage level of 60–64-y-old age group (23.9%) 5 y after introduction of RZV.

^d^All diseases assume waning occurs linearly from the base-case value to zero over the duration of protection period. See Supplement Table S6 for additional details on waning assumption in the base case. For sensitivity analyses, the lower and upper bound scenarios tested assumed linear waning similar to the base-case analysis.

### Modeled population

The modeled population is aligned with the model by McLaughlin et al.,^1^ focusing on the same four vaccine-preventable diseases among the same population of US adults aged 50 y and older; this population was also included in a subgroup analysis by Ozawa et al.^2^

**Figure 1.**
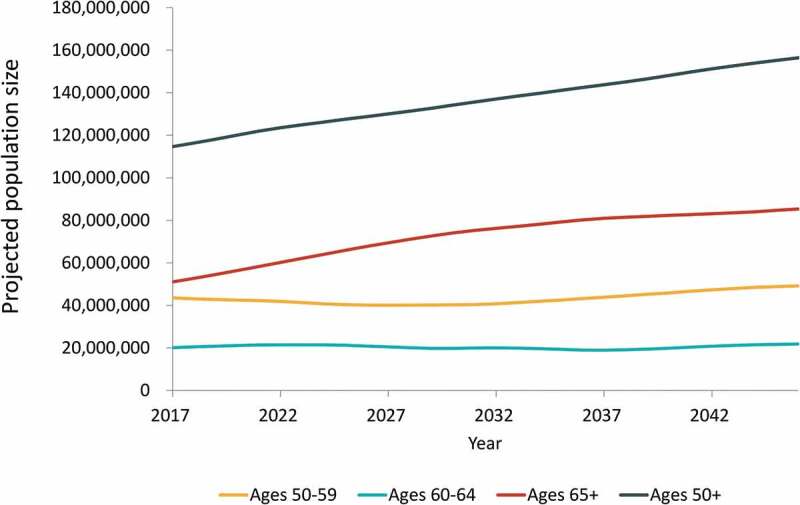
Projected United States population size over 30-y time horizon, by age group (years)

Note: Population projections were taken directly from the US Census Bureau projections for the years 2017 through 2046.^11^https://www.census.gov/data/datasets/2014/demo/popproj/2014-popproj.html

In Year 1 (2017), the model starts with the US population of adults aged 50 y and older (stratified by 1-y age groups) and applies estimated disease incidence among unvaccinated individuals, as well as current estimated levels of vaccine coverage and efficacy to estimate the number of cases of each disease. In each subsequent year, projected estimates of the US population are used correspondingly to calculate numbers of cases of disease among individuals aged 50 y and older, holding all other inputs constant over time. By using these population projections, the model accounts for the aging of the population, as well as projected changes in all-cause mortality and net migration in each year. This approach also allows a new cohort of individuals who have just turned 50 y to “age into” the model in each year. Figure 1 illustrates the projected population changes over time.

### Vaccine coverage and effectiveness

Vaccines currently recommended by the ACIP for adults aged 50 y and older are presented in Table 2.^3^

**Table 2.** Current vaccine recommendations for adults aged 50 y and older

Vaccine	Recommendation
Influenza	One dose of seasonal influenza vaccine each year
Tdap	One dose of Tdap vaccine (followed by a booster^a^ every 10 y)
Zoster	Two doses of RZV, including those individuals who were previously vaccinated with ZVL (preferred); or 1 dose of ZVL
Pneumococcal	One dose of PCV-13 (based on a shared clinical decision between health care provider and patient) and one dose of PPSV-23 for those adults aged 65 y and older

HZ, herpes zoster; PCV-13, pneumococcal conjugate vaccine; PPSV-23, pneumococcal polysaccharide vaccine; RZV, recombinant zoster vaccine; Td, tetanus-diphtheria; Tdap, tetanus, diphtheria, and acellular pertussis; ZVL, zoster vaccine live.

^a^In October 2019, ACIP voted to revise the Td recommendation for decennial booster vaccination, allowing providers to administer either Td or Tdap every 10 y throughout life to ensure protection against tetanus and diphtheria.

For annual influenza vaccination, the model allows for a certain percentage of individuals aged 50 y or older to be vaccinated each year based on annual age-specific vaccine coverage rates from the National Immunization Survey and the Behavioral Risk Factor Surveillance System, using average coverage values over the past seven seasons (2010/2011 to 2016/2017).^40^ Influenza vaccine protection is assumed to last one season and vaccine waning is not considered.

For pertussis, HZ, and pneumococcal vaccines, the model estimates vaccine effectiveness using durations of protection and waning curves based on data from the published literature. These assumptions are summarized in Supplementary Table S6. For these vaccines, coverage data from the 2016 National Health Interview Survey are reported as the percentages of people who have ever received vaccination (for HZ and pneumococcal vaccines) or the percentage of people who have received vaccination in the last 10 y (for Tdap vaccine).^4^ To account for these data and the assumption that vaccination coverage remains constant over time, the model uses an incremental approach to estimate the number of individuals vaccinated in a given year; the number of individuals “ever vaccinated” or “vaccinated in the last 10 years” in the modeled year are subtracted by the number of individuals estimated from the previous year, adjusting for deaths of vaccinated individuals. Each year, the model then calculates the proportion of vaccinated individuals with full or waned protection based on the number of individuals “ever vaccinated” or “vaccinated in the last 10 years” and the number of individuals vaccinated in the given year.

For pertussis vaccination, the model assumes that individuals aged 50 y and older who were previously vaccinated with Tdap in the last 10 y were uniformly distributed across the 10-y time frame, not taking into account changes in age recommendations over time (i.e., ACIP expanded the recommendation for Tdap to individuals aged 65+ y in 2012). Our analysis focuses on the incremental protection provided by Tdap vaccine against pertussis compared with protection provided by tetanus diphtheria (Td) vaccine and does not include the burden of tetanus and diphtheria. As a simplifying assumption, the model also does not include the recommended booster doses every 10 y with either Td or Tdap vaccine. For HZ vaccination, the model assumes that individuals aged 60 y and older previously received the existing zoster vaccine live (ZVL) beginning in 2006 (based on date of licensure and ACIP recommendations^41,42^) with uniformly distributed uptake between 2006 and 2017. Beginning in Year 2 (2018), the model assumes that individuals aged 50 y and older received recombinant zoster vaccine (RZV, based on ACIP recommendations^3,43^ and date of licensure^44^), regardless of whether they had previously been vaccinated with ZVL. Of those vaccinated with RZV, 69% were assumed to receive the second dose.^45^ For pneumococcal vaccination, the model assumes that individuals aged 65 y and older began receiving both the conjugate and polysaccharide vaccines in 2014 (consistent with ACIP recommendations^42^) with uniformly distributed uptake between 2014 and present to calculate waning. Reported vaccine coverage for those who had “ever had a pneumonia shot” was tested in sensitivity analysis to test lower values for the percentage receiving both vaccines. For simplicity, the model excludes vaccination of high-risk individuals younger than 65 y of age.

### Model analyses and validation

For each disease, the model calculates both annual health outcomes (i.e., number of disease cases overall and by severity, disease-related deaths) and undiscounted direct medical and societal perspective costs. Vaccination costs are not included. For the societal perspective, costs include direct medical costs incurred by payers and by patients (i.e., costs of over-the-counter medications), as well as disease-related indirect costs (i.e., productivity loss costs due to disease cases and mortality). The focus for this burden of disease analysis is on undiscounted results to appropriately capture the projected *actual* outcomes and costs to society, as well as to enable comparison with the undiscounted results previously reported by McLaughlin et al.^1^ and Ozawa et al.^2^ however discounted costs were also calculated.

The human capital approach was used to estimate productivity loss costs due to disease-related mortality for each of the four modeled diseases, using mean annual age-specific market and nonmarket productivity estimates and age-specific estimates of remaining life expectancy.^46–48^

It is assumed that individuals can develop only one case of disease each year. Although some of the modeled diseases have long-term complications that may incur costs over multiple years (e.g., postherpetic neuralgia [PHN] for HZ, long-term neurological sequelae for pneumococcal disease), only costs in the first year of the disease are included.

The annual and cumulative disease burden is calculated overall and by age (50–59, 60–64, 65+ y) over a time horizon of up to 30 y. In addition to base-case analyses, one-way sensitivity analyses were conducted for several model input parameters, varying each parameter up and down by a clinically plausible range (e.g., 95% confidence interval) to assess the impact on total cost burden of disease. These clinically plausible ranges (Table 1) were also used in a post-hoc multiway sensitivity analysis in which vaccination coverage, efficacy, duration of protection, and disease incidence were varied simultaneously to generate lower and upper bounds around the overall burden of disease estimates. To validate the underlying model structure and inputs, the 1-y burden of disease estimates were compared to those reported previously by McLaughlin et al. and Ozawa et al.^1,2^ Additional details on model validation can be found in Supplementary Material S-3.

## Results

### Base-case analysis results

**Figure 2.**
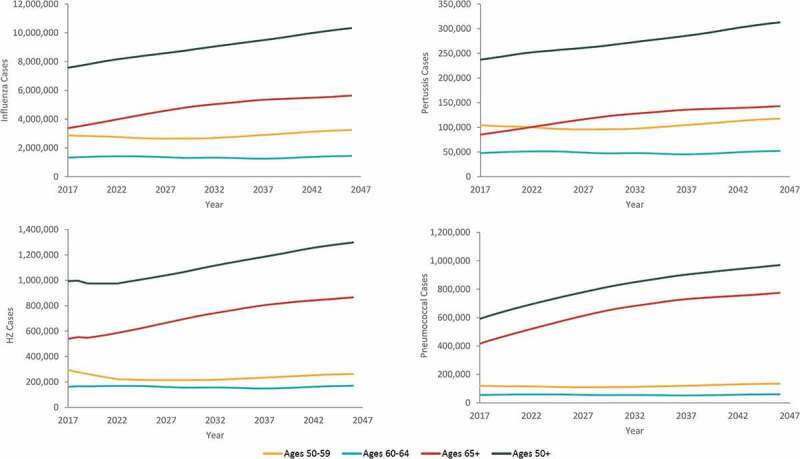
Projected annual (undiscounted) cases of influenza, pertussis, herpes zoster (HZ), and pneumococcal disease by age group, from 2017–2046

Note: The age groups presented in this figure (50–59 y, 60–64 y, and 65+ y) are different sizes.

Over a 30-y period, the projected annual (undiscounted) number of cases of influenza, pertussis, HZ, and pneumococcal disease show trends of substantial growth (36%, influenza; 32%, pertussis; 31%, HZ; 64%, pneumococcal disease), driven primarily by the 65+ y age category (Figure 2). Growth in annual direct medical costs over the study period (49%, influenza; 46%, pertussis; 43%, HZ; 61%, pneumococcal disease) was higher as compared with the growth in projected annual cases of disease. Across all four diseases, annual undiscounted direct medical costs are projected to increase from approximately 17 USD billion in Year 1 (Supplementary Table S8) to nearly 26 USD billion in Year 30. From the societal perspective, annual costs for all four diseases are projected to increase from approximately 35 USD billion in Year 1 to nearly 49 USD billion in Year 30. These increases in costs represent a 53% increase in annual direct medical costs and a 40% increase in annual societal costs over the 30-y period. In contrast, the size of the US population aged 50 y and older is projected to only increase by 36% over the period (from 115 million individuals in Year 1 to 156 million individuals in Year 30) (Figure 1).

**Figure 3.**
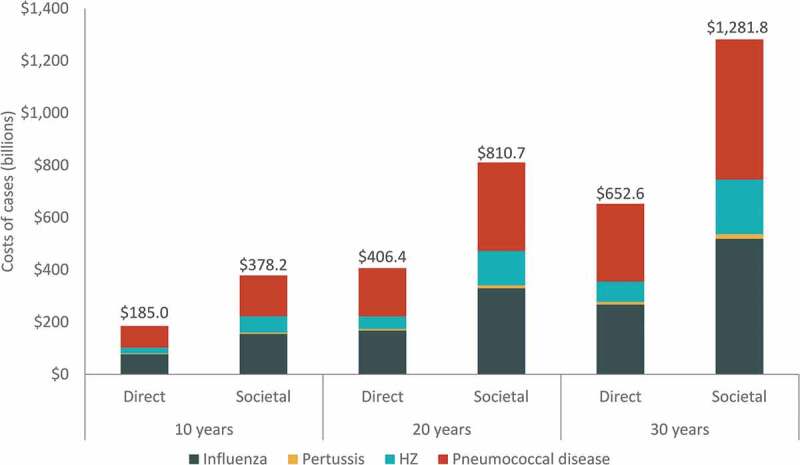
Cumulative total direct and societal costs of cases over 10-y, 20-y, and 30-y time horizons, by disease (billions, USD)

HZ, herpes zoster; USD, United States dollars. Note: All costs are in 2018 USD and are presented undiscounted.

The cumulative burden of disease over the 30-y period is estimated to include approximately 270 million cases of influenza, 8 million cases of pertussis, 33 million cases of HZ, and 25 million cases of pneumococcal disease (Table 3). The four diseases are projected to result in over 1 million disease-related deaths over the 30-y period. Combined cumulative undiscounted direct medical costs for all four diseases are projected to increase from 185 USD billion over 10 y to nearly 653 USD billion over 30 y, with influenza and pneumococcal disease comprising the majority of costs (Figure 3). From the societal perspective, combined cumulative costs increase from 378 USD billion over 10 y to 1.3 USD trillion over 30 y, with pneumococcal disease comprising approximately 42% and influenza comprising 40% of all costs over 30 y. A detailed breakdown of the 30-y projected disease burden and costs (undiscounted or discounted) by disease, age group, and disease severity can be found in Supplementary Tables S9 and S10.

**Table 3.** Cumulative 30-y burden of disease (undiscounted), by age and overall

Disease/outcomes	Age (years)			
	50–59	60–64	65+	Total
**Influenza**				
Number of cases	85,647,572	40,497,341	143,743,003	269,887,916
Number of disease-related deaths	19,122	9,041	413,431	441,594
Total direct medical costs (paid by payer)	$45,883,123,737	$21,695,238,548	$199,334,983,446	$266,913,345,731
Total societal costs	$130,638,982,255	$57,180,426,644	$330,237,620,136	$518,057,029,035
**Pertussis**				
Number of cases	3,099,362	1,464,449	3,646,532	8,210,343
Number of disease-related deaths	800	378	3,763	4,941
Total direct medical costs (paid by payer)	$2,095,969,820	$990,345,974	$7,025,829,059	$10,112,144,854
Total societal costs	$5,244,058,657	$2,285,989,591	$10,463,245,809	$17,993,294,058
**Herpes zoster**				
Number of cases	7,088,507	4,806,332	21,559,476	33,454,315
Number of disease-related deaths	0	0	0	0
Total direct medical costs (paid by payer)	$8,707,018,103	$9,957,663,481	$58,880,235,215	$77,544,916,799
Total societal costs	$34,780,702,024	$34,068,701,432	$140,002,845,262	$208,852,248,718
**Pneumococcal disease**				
Number of cases	3,565,219	1,685,768	19,340,814	24,591,801
Number of disease-related deaths	56,226	26,586	595,442	678,253
Total direct medical costs (paid by payer)	$50,706,297,657	$23,975,813,631	$223,336,228,923	$298,018,340,210
Total societal costs	$123,582,512,818	$44,936,386,159	$368,416,538,827	$536,935,437,804
**Total burden of disease**				
Number of disease-related deaths	76,147	36,005	1,012,636	1,124,788
Total direct medical costs (paid by payer)	$107,392,409,318	$56,619,061,633	$488,577,276,643	$652,588,747,594
Total societal costs	$294,246,255,754	$138,471,503,826	$849,120,250,034	$1,281,838,009,614

USD, United States dollars.

Note: All cost outcomes are in 2018 USD and are presented undiscounted.

**Figure 4.**
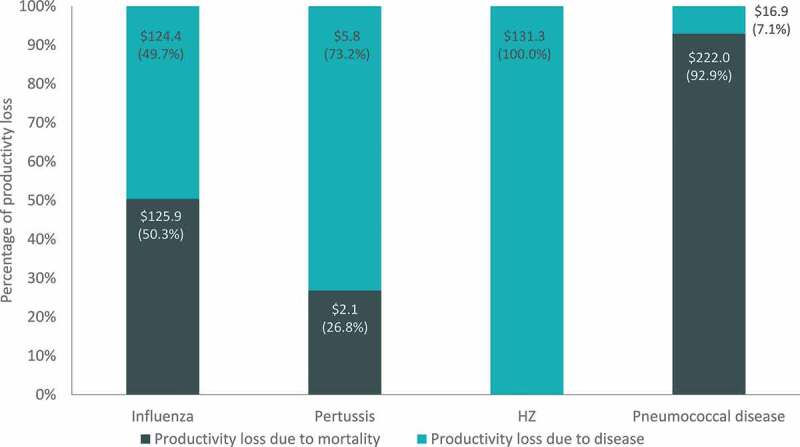
Cumulative 30-y productivity loss costs by disease (billions, USD)

HZ, herpes zoster; USD, United States dollars. Note: All costs are in 2018 USD and are presented undiscounted.

Projected burden of disease estimates and drivers of costs varied for each disease. For example, productivity loss costs due to disease morbidity and mortality ranged from approximately 8 USD billion for pertussis to almost 240 USD billion for pneumococcal disease (Figure 4). For influenza and pneumococcal disease, the majority of productivity loss costs are due to disease-related mortality (approximately 50% and 93%, respectively). In the case of pertussis and HZ (both diseases with a low risk of death), productivity losses are primarily a consequence of disease-related morbidity (approximately 73% and 100%, respectively).

Costs of higher severity cases represented a significant portion of overall direct costs for all modeled diseases. For example, over 30 y, hospitalized influenza cases made up approximately 33% of all influenza cases, while associated direct costs of hospitalized cases (~200 USD billion) accounted for over 75% of all direct costs for treatment of influenza. Regarding HZ, over 20% of cases were considered complicated (with PHN or other non-pain complications) but made up over 58% of all direct medical costs for treating HZ. Inpatient pneumococcal cases represented only 37% of pneumococcal disease cases but contributed to over 85% of the direct costs attributed to the disease. Finally, this trend is also observed with pertussis, where the majority of direct costs were attributed to severe cases (over 80%) even though they only accounted for 7% of all cases (Supplementary Table S9).

### One-way sensitivity analysis results

**Figure 5.**
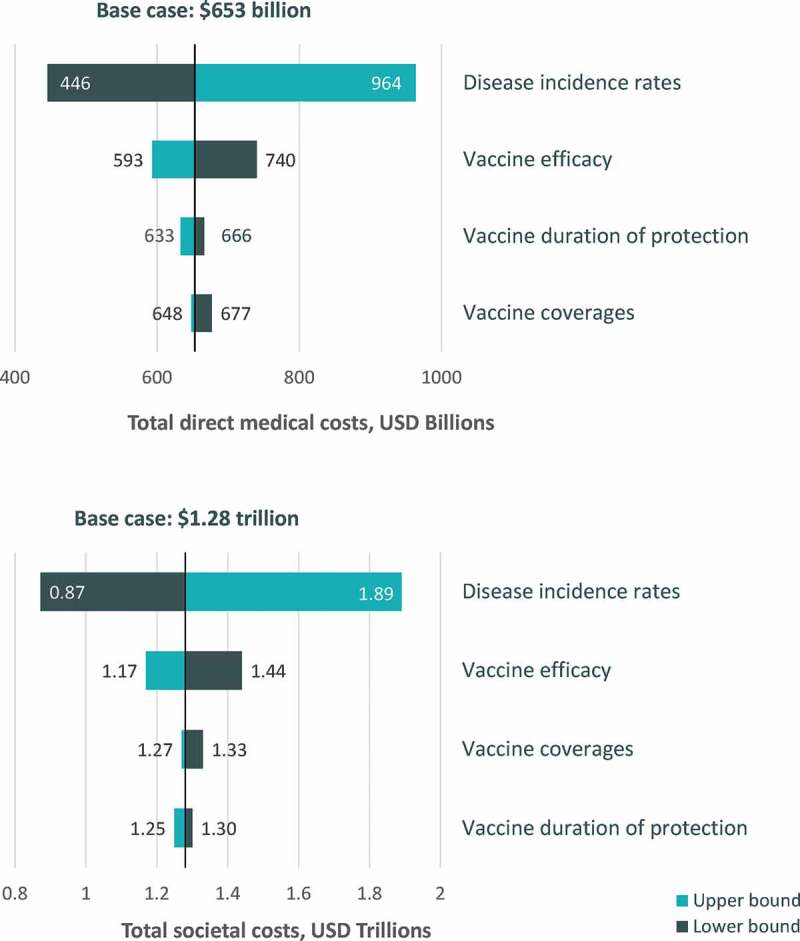
One-way sensitivity analysis results for cumulative 30-y burden of disease

When clinically plausible ranges were explored for key input variables in one-way sensitivity analysis, the cumulative 30-y burden of disease from both the direct medical costs and societal perspective was most sensitive to changes in disease incidence (Figure 5). Overall, the cumulative 30-y burden of disease varied from 446 USD billion to 964 USD billion for the direct medical cost perspective and from 872 USD billion to 1.9 USD trillion for the societal cost perspective across all varied model assumptions. The burden of disease estimates were less sensitive to changes in assumptions regarding vaccine coverage, duration of protection, and efficacy.

USD, United States dollars.

### Multiway sensitivity analysis results

**Figure 6.**
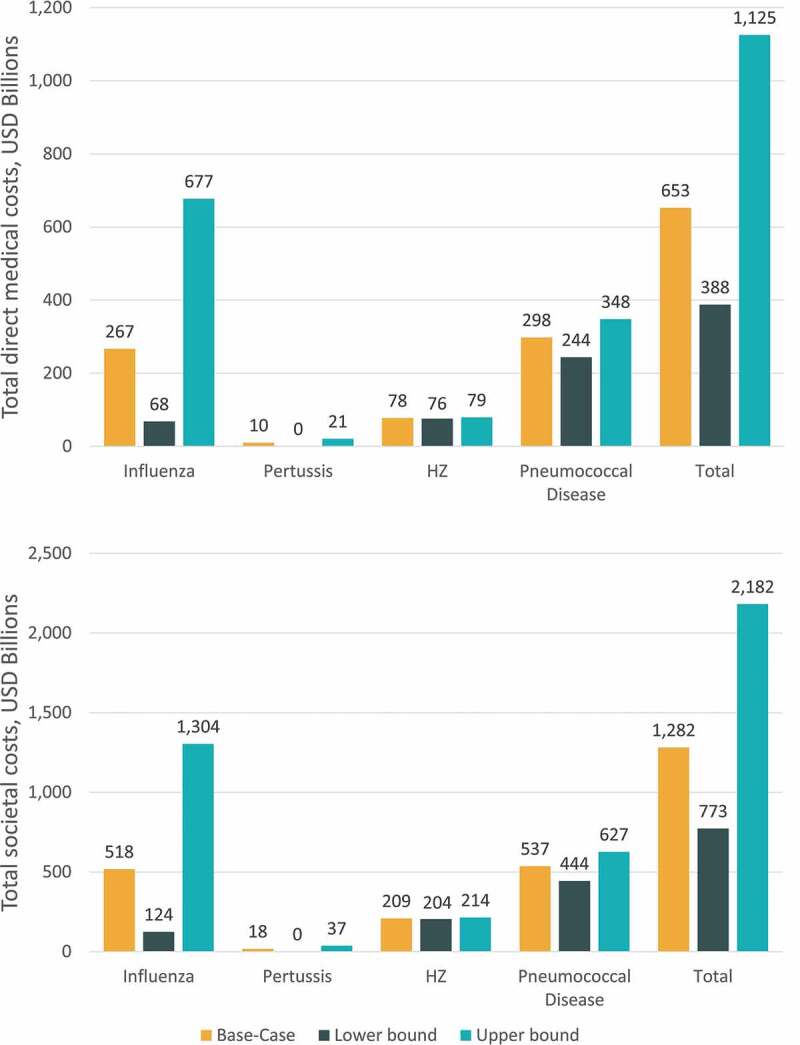
Multiway sensitivity analysis results for cumulative 30-y burden of disease

When inputs for disease incidence, vaccination coverage, efficacy, and duration of protection were varied simultaneously using clinically plausible ranges, the projected burden of disease over a 30-y time horizon ranged from 388 USD billion to 1.1 USD trillion for the direct medical cost perspective and from 773 USD billion to 2.2 USD trillion for the societal perspective (Figure 6). Multiway sensitivity results were most variable for influenza, ranging from 68 USD billion to 677 USD billion for the direct medical cost perspective and from 124 USD billion to 1.3 USD trillion for the societal perspective (Figure 6).

USD, United States dollars. Note: The lower bound disease burden results were estimated by using the lower bound ranges for disease incidence and using the upper bound ranges for vaccine coverage, vaccine efficacy, and duration of protection. The upper bound disease burden results were estimated by using the lower bound ranges for vaccine coverage, vaccine efficacy, and duration of protection and using the upper bound ranges for disease incidence. The pertussis lower bound burden is displayed as zero due to rounding; the actual value is ~0.1 USD billion from the direct medical costs perspective and ~0.2 USD  billion from the societal perspective.

## Discussion

Over a 30-y period, the projected burden of influenza, pertussis, HZ, and pneumococcal disease in adults aged 50 y and older from the current study is substantial. The model predicted approximately 270 million cases of influenza, 8 million cases of pertussis, 33 million cases of HZ, and 25 million cases of pneumococcal disease, resulting in a cumulative total undiscounted cost of approximately 653 USD billion (425 USD billion discounted) from the direct cost perspective and 1.3 USD trillion (over 800 USD billion discounted) from the societal perspective. In the base-case analysis, the model assumed that disease incidence, disease-related mortality, vaccination coverage, and vaccine efficacy all remained constant; however, the projected annual number of cases of each disease grew over the 30-y period, driven by increases in population size due to the aging of the US population. Increases in the annual economic burden of disease from Year 1 to Year 30 (53% increase in direct costs and 40% increase in societal costs) outpaced the rate of overall growth in the population aged 50 y and older (36% increase). These results demonstrate a shift of the US population toward age groups that are more susceptible to disease and disease-related morbidity and mortality.

Lower and upper bound estimates of disease burden from the one-way and multiway sensitivity analyses highlight the substantial burden of disease expected across the range of clinically plausible inputs for disease incidence, vaccine coverage, efficacy, and duration of protection. Results were most sensitive to model inputs related to disease incidence, as demonstrated by the one-way sensitivity analysis results. These results were driven primarily by the highly variable disease incidence across influenza seasons, with the lowest and highest incidence observed across seasons ranging nearly 10-fold. Although the projected burden of disease estimates have a high level of uncertainty over the 30-y time horizon (ranging approximately 2–3 fold in magnitude for both the direct and societal perspectives), the projected cost of vaccine-preventable diseases is substantial even at the lower bound estimates.

The projected increase in vaccine-preventable disease burden among adults aged 50 y and older causes concern for the sustainability of existing health care provision over time.^5^ For influenza alone, our model predicts that cases will result in approximately 8 million hospitalizations, 4 million emergency department visits, and 107 million outpatient visits over the next several decades, which will need to be accommodated by health care facilities and providers. Efforts to reduce disease incidence and/or severity by policies aimed at increasing vaccination coverage and/or effectiveness of vaccines through research and funding priorities, as well as education of both medical providers and the general population on the importance and value of vaccination as a life-course approach, are needed to reduce the potential strain on the health care system and increase the population wellbeing and productivity over the next 30 years.

Although the CDC and the Department of Health and Human Services provide information on strategies to increase adult vaccination rates (e.g., through immunization quality improvement projects and/or reminder systems),^49,50^ low vaccination rates among adults persist. Historically, vaccine coverage rates in the US have been lower among adults compared to children.^4,51^ This is likely due to a variety of factors that promote childhood vaccination, including state laws requiring vaccination for school entry and the federally funded Vaccines for Children Program.^49^ Lack of regularly scheduled well visits for adults, akin to “well child” visits that correspond with CDC recommendations for childhood vaccinations, may also be a differentiating factor; utilization of Medicare’s Annual Medicare Wellness Visit is associated with likelihood of vaccination.^52^ Patient out-of-pocket costs are another barrier to vaccination in older adults. Medicare beneficiaries may face significant co-pays or co-insurance for vaccines covered under Medicare Part D (e.g., HZ and Tdap). Multiple studies have shown an association between increased patient out-of-pocket responsibility and reversal of vaccination claims (a proxy for no vaccination), both at the physician’s office and in the pharmacy setting.^53–55^

Implementation of additional quality measures for adult vaccination may be one solution to elevate the status of adult vaccination as an essential component of preventive care, much like childhood vaccination. While measures for adult influenza and pneumococcal vaccination exist in many quality measurement programs,^56^ a composite measure assessing compliance with all age-based recommended vaccines would be useful in monitoring overall adult vaccine program effectiveness.^57^ A composite vaccine measure including influenza, pneumococcal, Tdap, and HZ vaccines was successfully tested in clinics affiliated with the Indian Health Service,^57^ and an adult composite measure is currently being voluntarily reported in the 2020 HEDIS (Healthcare Effectiveness Data Information Set) measure set.^58^

In addition to improving vaccination coverage of currently available adult vaccines, further research and development efforts to improve vaccine effectiveness could also help reduce the projected burden of disease moving forward. Immunosenescence, the gradual deterioration of the immune system brought on by aging, results in older adults having less robust immune responses to vaccines.^59^ The use of adjuvants, substances added to enhance immunogenicity, in vaccines for influenza^60^ and HZ^33^ have demonstrated potential for improving vaccine effectiveness in older adults. Additionally, research efforts to improve the durability of vaccine protection, such as those by the National Institute of Allergy and Infectious Diseases,^61^ the European Union,^62^ and private companies in pursuit of a “universal” influenza vaccine covering multiple influenza strains over multiple influenza seasons increase the potential to reduce disease burden, even at current vaccine coverage rates.

The goal of this study was to use a consistent modeling framework across the four vaccine-preventable diseases to estimate the projected burden of disease over time at the population level. To maintain a simple model structure, population dynamics were implicitly captured by using cross-sectional projected population estimates from the US Census Bureau^11^ over the modeled time horizon. To meet the study objectives, the model analyses were conducted at the population level and did not involve additional complexities of tracking individual, single-year-of-age cohorts over time or modeling dynamic disease transmission. To estimate the effects of population aging on disease burden, the model also held other input parameter values constant over the modeled time horizon (e.g., assuming no changes to age-specific disease incidence rates, disease-related mortality rates, vaccination coverage, vaccine efficacy, and costs).

To our knowledge, this is the first study to use a consistent modeling framework across diseases to project the burden of vaccine-preventable diseases among adults aged 50 y and older based solely on projected changes in the US population. Other studies have estimated the projected burden of HZ^5^ and pneumococcal disease^6^ over time but with several key differences as compared with our model. The model by Varghese et al.^5^ projected the potential burden of HZ in the US in the year 2030 by taking into consideration both trends in HZ incidence and population aging, but projections were based on HZ incidence rates before HZ vaccines became available. Wroe et al.^6^ projected the potential burden of pneumococcal pneumonia in the US between 2004 and 2040 based on projected population growth and shift in age distribution. However, this analysis only included pneumococcal pneumonia and did not consider all pneumococcal disease. Additionally, incidence data captured in the Wroe et al. model were from 2004, prior to the introduction of routine infant vaccination with PCV-13 that led to notable decreases in incidence among unvaccinated children and adults.

The model structure excluded some disease-specific details included in previous economic models (e.g., herd immunity, serotype replacement, long-term complications, cross-protection from previous influenza vaccinations) and did not account for pandemics or outbreaks. Although the input parameter values for pneumococcal disease implicitly captured costs and disease incidence for high-risk and low-risk populations aged 65 y and older, the input values and results were not modeled separately for these populations. The four vaccine-preventable diseases were also modeled over time separately and did not allow for interaction across the diseases (e.g., pneumococcal disease-related deaths were not accounted for within the modeled populations for the other diseases).

The model used aggregate-level vaccine coverage and average vaccine effectiveness values and waning functions that were not specific to vaccine products. For example, the effectiveness and cost of influenza vaccines represent an average across the available seasonal vaccines for adults. Similarly, for pertussis and pneumococcal vaccines, the effectiveness and costs are an average across products. The exception is HZ vaccines, where effectiveness and cost of ZVL are modeled in Year 1 (2017) and effectiveness and cost of RZV are modeled in subsequent years. These simplifying assumptions were made where the impact on the cost and clinical projections were deemed minor, while keeping the model structure and parameterization robust enough to address study objectives.

In 2019 ACIP made two relevant updates to the adult vaccination recommendations. In June 2019, ACIP voted to revise the pneumococcal vaccination recommendation for healthy individuals over 65 y of age.^63^ While pneumococcal polysaccharide vaccine (PPSV-23) is still universally recommended, it is now recommended that the decision to give PCV-13 be made jointly between physician and patient. This change in PCV-13 recommendation was made in part due to the waning of PCV-13-serotype disease in the older adults after implementation of a universal pediatric recommendation, and from minimal population-level decreases in pneumonia and invasive pneumococcal disease since the adult recommendation in 2014. Results of our analysis may underestimate the burden of pneumococcal disease in adults aged 65 y and older if use of PCV-13 decreases substantially in the future. However, our analysis likely overestimates the burden of pneumococcal disease at younger ages because of the exclusion of pneumococcal vaccination in high-risk adults 50–64 y of age. Additionally, in October 2019, ACIP voted to revise the Td recommendation for decennial booster vaccination, allowing providers to administer either Td or Tdap every 10 y throughout life to ensure protection against tetanus and diphtheria and the option to also protect against pertussis.^64^ This change in Td recommendation was made to follow the widespread practice of administering Tdap instead of Td for booster vaccinations. Because the recommendation changed during the time frame of our model and booster doses were not modeled in the current analysis, our results may overestimate the burden of pertussis over the 30-y time horizon; however, the overestimation of the total burden of disease is likely to be relatively small given the small proportion (~1-2%) pertussis contributes to the overall burden.

## Conclusion

Maintaining current vaccine coverage rates among older adults is projected to lead to notable increases in influenza, pertussis, HZ, and pneumococcal disease burden over the next several decades due to population aging. Corresponding increases in the economic burden and health care resource utilization required to care for these vaccine-preventable diseases may cause increasing strain on the US health care system.

## Supplementary Material

Supplemental Material
