# Supplementary material for: Impact of population aging on the burden of vaccine-preventable diseases among older adults in the United States
Source: Hum Vaccin Immunother. 2020 Aug 6;17(2):332–43. doi: 10.1080/21645515.2020.1780847 (PMC7899694; doi:10.1080/21645515.2020.1780847)
Supplement: Supplemental Material [file KHVI_A_1780847_SM0431.pdf]

Supplemental Material

Table of Contents

S-1: Disease-Specific Decision Tree Model Structures

S-2: Model Input Parameter Values

S-3: Projected Population Size Over Modeled Time Horizon

S-4: Model Validation of 1-Year Burden of Disease Results

S-5: Detailed 30-Year Burden of Disease Analysis Results

S-1: Disease-Specific Decision Tree Model Structures

Figure S1. Influenza Decision Tree Model Structure

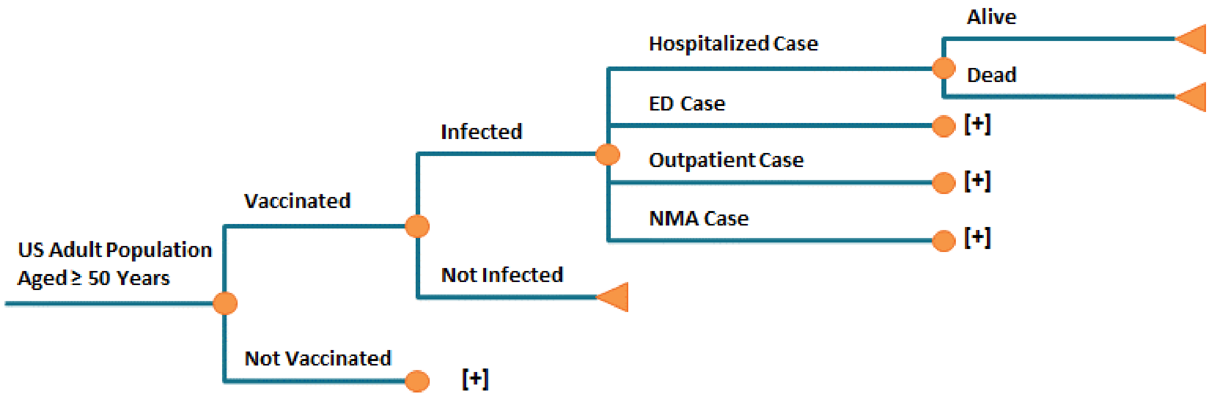

ED, emergency department; NMA, nonmedically attended.

[+] Indicates clinical pathway is the same as above.

Figure S2. Pertussis Decision Tree Model Structure

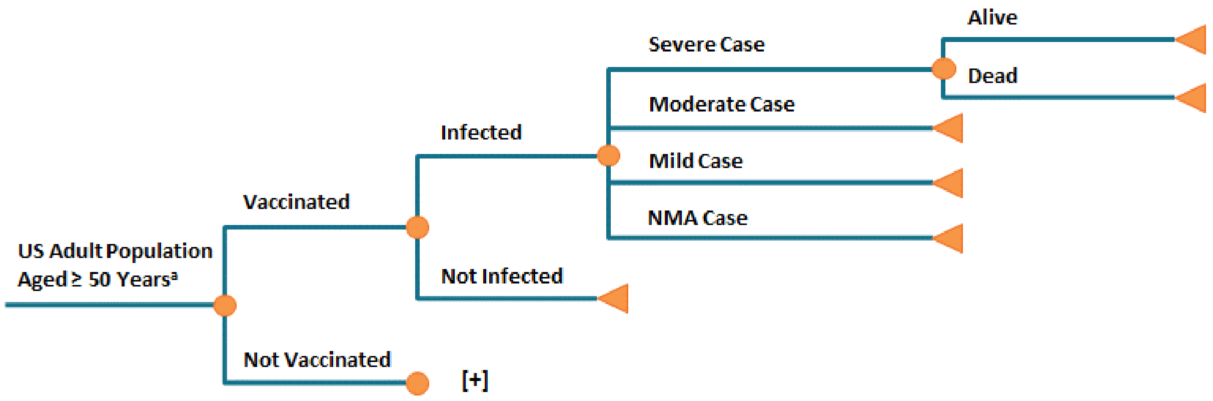

NMA, nonmedically attended; Tdap, tetanus, diphtheria, and acellular pertussis.

[+] Indicates clinical pathway is the same as above.

[a] Only individuals not previously vaccinated with Tdap in the last 10 years are included in the eligible population each year.

Figure S3. Herpes Zoster Decision Tree Model Structure

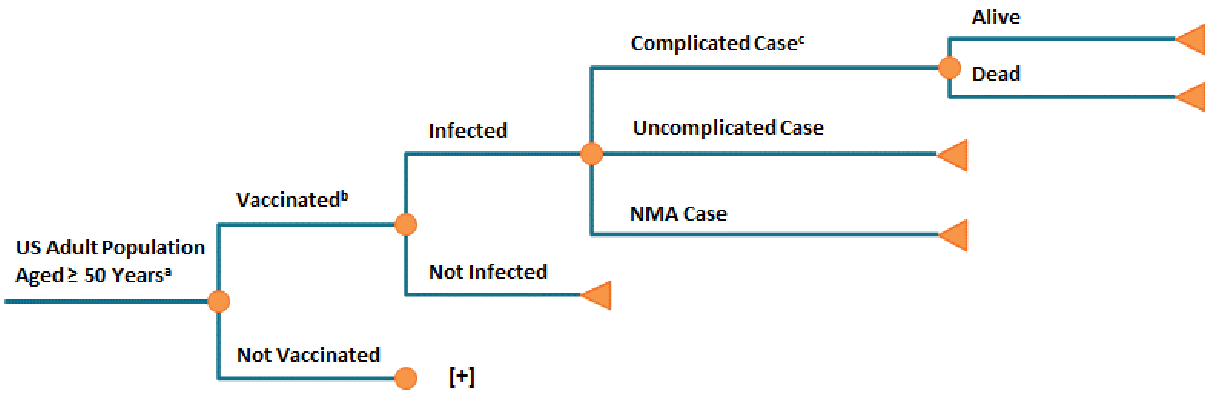

NMA, nonmedically attended; PHN, postherpetic neuralgia; RZV, recombinant zoster vaccine, adjuvanted; ZVL, zoster vaccine live.

[+] Indicates clinical pathway is the same as above.

[a] Individuals not previously vaccinated with RZV are included in the eligible population each year (i.e., in 2017, everyone is eligible).

[b] In Year 1 (2017), adults aged 60 years or older are assumed to be vaccinated with ZVL. In Year 2 onward, adults aged 50 years or older are assumed to be vaccinated with RZV.

[c] Complicated with PHN and/or non-pain complications.

Figure S4. Pneumococcal Disease Decision Tree Model Structure

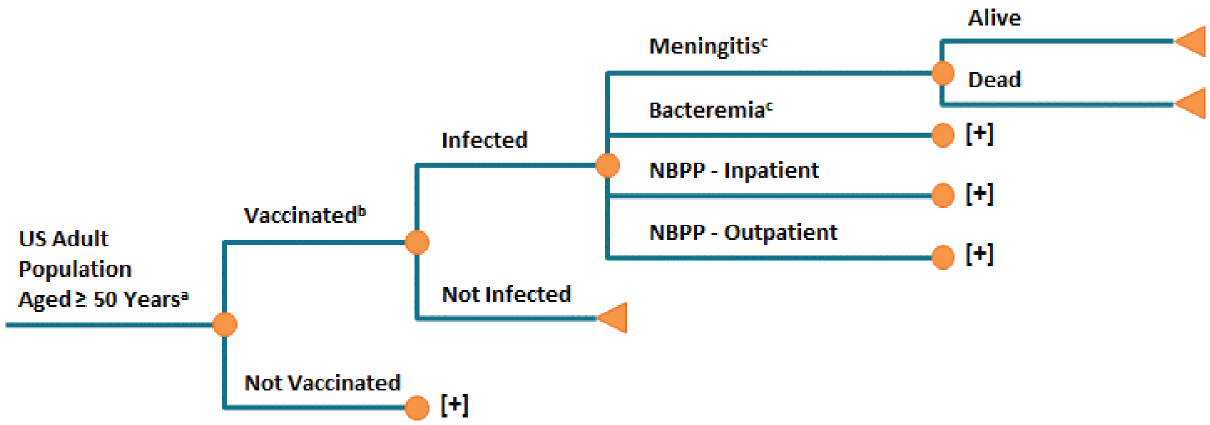

ACIP, American Committee on Immunization Practices; NBPP, nonbacteremic pneumococcal pneumonia; PCV-13, pneumococcal conjugate vaccine; PPSV-23, pneumococcal polysaccharide vaccine.

[+] Indicates clinical pathway is the same as above.

[a] Only individuals who have not received any pneumococcal vaccine are included in the eligible population each year.

[b] Only adults aged 65 years and older are vaccinated, with both PCV-13 and PPSV-23, as recommended by ACIP as part of routine vaccination or in consultation with health care providers.

[c] All cases are assumed to be medically treated in an inpatient (i.e., hospitalized) setting.

S-2: Model Input Parameter Values

Table S1. Disease-Specific Input Parameter Values: Influenza

Model Parameter	Ages 50-59 Years	Ages 60-64 Years	Ages 65+ Years	Sources and Notes
Incidence per 100,000 among unvaccinated	8,218	8,218	9,264	Kostova et al.,[1] Bresee et al.,[2] Reed et al.,[3] and CDC surveillance data[4,5] used to estimate incidence of influenza cases in the absence of vaccination; average across 9 seasons (2005/2006 to 2015/2016, excluding 2009/2010 due to emergence of the H1N1 virus and 2011/2012 due to lack of data in sources for that year)
Case-severity distribution				
Hospitalized case	0.7%	0.7%	4.9%	Talbird et al.[7] (citing Kostova et al.[1]; Bresee et al.;[2] Reed et al.;[3] and CDC[4] [using the same methodology as Bresee et al.[2] and Reed et al.[3]])
ED case	2.6%	2.6%	0.5%	Talbird et al.[7] (citing AHRQ HCUPnet[6] [2006, 2007, 2008, 2009, 2011, 2013]; mean of 6 years of AHRQ HCUPnet data)
Outpatient case	35.8%	35.8%	42.8%	Talbird et al.[7] (calculated as the residual of percentage hospitalized and percentage of ED cases among all medically attended cases)
NMA case	60.9%	60.9%	51.8%	Talbird et al.[7] (calculated as the complement of percentage medically attended based on data from Kostova et al.[1]; Bresee et al.;[2] Reed et al.[3] and CDC[44,5])
Percentage of cases resulting in death				Talbird et al.[7] (derived from Reed et al.[8] using mortality rates for ages 18-64 and 65+ years over 3 seasons [2010/2011, 2011/2012, 2012/2013])
Hospitalized case	2.5%	2.5%	3.9%	
Nonhospitalized case (i.e., ED, outpatient, or NMA case)	0.005%	0.005%	0.101%	
Direct medical costs per case[a]				Hospitalized and outpatient costs: Molinari et al.;[9] weighted average of low-risk and high-risk patients<br>ED costs: Karve et al.;[10] total influenza-related ED visit costs among patients with at least 1 influenza-related ED visit; mean across 4 influenza seasons (2005/2006 to 2008/2009)
Hospitalized case	$46,620	$46,620	$23,108	
ED case	$362	$362	$362	
Outpatient case	$559	$559	$590	
NMA case[b]	$0	$0	$0	Assumption
Indirect costs per case[a]				

Model Parameter	Ages 50-59 Years	Ages 60-64 Years	Ages 65+ Years	Sources and Notes
Cost of productivity loss due to disease case	$715	$715	$238	Productivity losses for cases of disease taken from McLaughlin et al. ,[11] citing Grosse et al.[12]
Cost of productivity loss due to mortality from disease case	Varies by age (as shown in Supplemental Table 7)			Calculated by single year of age based on life expectancy (Arias et al.[13]) and mean annual market and non-market productivity for individuals aged 50 years and older (Grosse et al.[14])

AHRQ, Agency for Healthcare Research and Quality; CDC, Centers for Disease Control and Prevention; CPI, Consumer Price Index; ED, emergency department; HCUP, Healthcare Cost and Utilization Project; NMA, nonmedically attended; OTC, over-the-counter; USD, United States dollars.

[a] All disease-related costs were inflated to 2018 USD using the medical care component of the CPI ,[15] when necessary. All productivity loss costs were inflated to 2018 USD using the employment cost index.[16]

[b] For the societal perspective, the model assumes a $5 OTC medication cost for NMA cases.

Table S2. Disease-Specific Input Parameter Values: Pertussis

Model Parameter	Ages 50-59 Years	Ages 60-64 Years	Ages 65+ Years	Sources and Notes
Incidence per 100,000 among unvaccinated	250.3[a]	250.3[a]	174.1	MMWR, Summary of Notifiable Diseases in the US, 2015 (most recent available; Adams et al.[17]); adjusted to estimate incidence among unvaccinated[b]; further adjusted by an underreporting factor of 100 (Chen et al.;[18] Masseria and Krishnarajah;[19] Acosta;[20] Millier et al.[21])
Case-severity distribution				
Severe case	3%	3%	12%	Based on hospitalization rates from Cortese et al.[22] appendix
Moderate case	86%	86%	74%	Percentage moderate cases calculated as the residual of severe and mild cases
Mild case	11%	11%	14%	Based on percentage without paroxysmal cough, see Cortese et al.[22] appendix
NMA case[c]	0%	0%	0%	Assumed 100% of cases are treated
Percentage of cases resulting in death				Severe case: Talbird et al.,[23] citing Caro et al.[24] Mild/moderate/NMA case: assumption; death among mild/moderate /NMA cases assumed to be negligible and not modeled
Severe case	0.86%	0.86%	0.86%	
Moderate/mild/NMA case	0.0%	0.0%	0.0%	
Direct medical costs per case[d]				
Severe case	$14,243	$14,243	$14,243	Acosta[20]
Moderate case	$280	$280	$280	
Mild case	$74	$74	$74	CMS;[25] cost of 1 physician visit (CPT code 99213)
NMA case[e]	$0	$0	$0	Assumption
Indirect costs per case[d]				
Cost of productivity loss due to disease case	$702	$702	$702	Productivity losses for cases of disease taken from McLaughlin et al.,[11] citing Grosse et al.[12]
Cost of productivity loss due to mortality from disease case	Varies by age (as shown in Supplemental Table 7)			Calculated by single year of age based on life expectancy (Arias et al.[13]) and mean annual market and non-market productivity for individuals aged 50 years and older (Grosse et al.[14])

CMS, Centers for Medicare and Medicaid Services; CPI, Consumer Price Index; CPT, Current Procedural Terminology; MMWR, Morbidity and Mortality Weekly Report; NMA, nonmedically attended; OTC, over-the-counter; Tdap, tetanus, diphtheria, and acellular pertussis; US, United States; USD, United States dollars.

[a] Based on data for ages 19-64 years.

[b] To estimate incidence of pertussis in the absence of vaccination, we applied Tdap effectiveness (70%) and observed vaccination coverage levels in 2015.

[c] This assumption that 100% of cases are treated is applied even when applying the underreporting factor (i.e., even cases that are not reported are assumed to be treated, with associated costs applied to the not reported cases).

[d] All disease-related costs were inflated to 2018 USD using the medical care component of the CPI,[15] when necessary. All productivity loss costs were inflated to 2018 USD using the employment cost index.[16]

[e] For the societal perspective, the model assumes a $5 OTC medication cost for NMA cases.

Table S3. Disease-Specific Input Parameter Values: Herpes Zoster

Model Parameter	Ages 50-59 Years	Ages 60-69 Years	Ages 70-79 Years	Ages 80+ Years	Sources and Notes
Incidence per 100,000 among unvaccinated	674	932	1,202	1,278	Johnson et al.[26] (Supplement Table)
Case-severity distribution					
Complicated case, with PHN or nonpain complications	12.1%	15.8%	26.5%	26.5%	Yawn et al.[27] (Table 3); assumed 100% of cases are care-seeking
Uncomplicated case	87.9%	84.2%	73.5%	73.5%	
NMA case	0.0%	0.0%	0.0%	0.0%	
Percentage of cases resulting in death	0%	0%	0%	0%	Assumption
Direct medical costs per case[a]					Yawn et al.[27] (Table 3)
Complicated case, with PHN or nonpain complications	$3,501	$7,164	$6,988	$6,988	
Uncomplicated case	$915	$1,113	$1,416	$1,416	
NMA case[b]	$0	$0	$0	$0	
Indirect costs per case[a]					
Cost of productivity loss due to disease case	$3,678	$5,017	$3,549	$3,338	Productivity losses for cases of disease taken from McLaughlin et al.,[11] citing Grosse et al.[12]
Cost of productivity loss due to mortality from disease case	Varies by age (as shown in Supplemental Table 7)				Calculated by single year of age based on life expectancy (Arias et al.[13]) and mean annual market and non-market productivity for individuals aged 50 years and older (Grosse et al.[14])

CPI, Consumer Price Index; NMA, nonmedically attended; OTC, over-the-counter; PHN, postherpetic neuralgia; USD, United States dollars.

[a] All disease-related costs were inflated to 2018 USD using the medical care component of the CPI,[15] when necessary. All productivity loss costs were inflated to 2018 USD using the employment cost index.[16]

[b] For the societal perspective, the model assumes a $5 OTC medication cost for NMA cases.

Table S4. Disease-Specific Input Parameter Values: Pneumococcal Disease

Model Parameter	Ages 50-64 Years	Ages 65-74 Years	Ages 75-84 Years	Ages 85+ Years	Sources and Notes
Incidence per 100,000 among unvaccinated[a]					IPD: CDC ABC Surveillance Report,[28] Emerging Infections Program Network, Streptococcus pneumoniae, 2013<br>NBPP: Stoecker et al.;[29] Simonsen et al.;[30] Nelson et al.;[31] 30% of all-cause pneumonia rates were assumed to be NBPP (Huang et al.;[32] Weycker et al.[33])
Total IPD	17.4	34.5	34.5	34.5	
Meningitis	1.1	2.2	2.2	2.2	
Bacteremia	16.3	32.3	32.3	32.3	
NBPP	257.5	1,015.6	1,015.6	1,015.6	
Pneumonia case-severity distribution[b]					Calculated based on data from Stoecker et al. ;[29] Simonsen et al.;[30] Nelson et al.[31]
Inpatient case	30.1%	40.6%	40.6%	40.6%	
Outpatient case	69.9%	59.4%	59.4%	59.4%	
Percentage of cases resulting in death					Meningitis and bacteremia: CDC ABC Surveillance Report,[28] Emerging Infections Program Network, Streptococcus pneumoniae, 2013<br>NBPP – inpatient: Stoecker et al.[29]<br>NBPP - outpatient: assumption
Meningitis/bacteremia	10.7%	14.9%	14.9%	14.9%	
NBPP - inpatient	3.2%	6.7%	6.7%	6.7%	
NBPP - outpatient	0.0%	0.0%	0.0%	0.0%	
Direct medical costs per case[c]					Stoecker et al.,[29] citing Truven Health Analytics MarketScan 2010 database
Meningitis/bacteremia	$45,789	$30,894	$30,894	$30,894	
NBPP - inpatient	$39,845	$26,560	$26,560	$26,560	
NBPP - outpatient	$145	$290	$290	$290	
Indirect costs per case[c]					
Cost of productivity loss due to disease case					Productivity losses for cases of disease taken from McLaughlin et al.,[11] citing Grosse et al.[12]
Meningitis/bacteremia	$3,206	$1,286	$855	$772	
NBPP - inpatient	$2,590	$1,039	$690	$625	
NBPP - outpatient	$1,233	$495	$329	$297	

Model Parameter	Ages 50-64 Years	Ages 65-74 Years	Ages 75-84 Years	Ages 85+ Years	Sources and Notes
Cost of productivity loss due to mortality from disease case	Varies by age (as shown in Supplemental Table 7)				Calculated by single year of age based on life expectancy (Arias et al.[13]) and mean annual market and non-market productivity for individuals aged 50 years and older (Grosse et al.[14])

ABC, Active Bacterial Core; CDC, Centers for Disease Control and Prevention; CPI, Consumer Price Index; IPD, invasive pneumococcal disease; NBPP, nonbacteremic pneumococcal pneumonia; US, United States; USD, United States dollars.

[a] Where possible, incidence data were from the post-PCV-7 or post-PCV-13 childhood vaccination introductions in the US. CDC (2013) Active Bacterial Core Surveillance data (2013 data) and inpatient NBPP data (2011-2012 data) represent post-PCV-7 and post-PCV-13 childhood vaccination. Most recent data available for outpatient NBPP are from 1998-2004 representing part of the post PCV-7 period in the US.

[b] Meningitis and bacteremia cases were assumed to be 100% inpatient cases.

[c] All disease-related costs were inflated to 2018 USD using the medical care component of the CPI,[15] when necessary. All productivity loss costs were inflated to 2018 USD using the employment cost index.[16]

Table S5. Age-Specific Vaccine Coverage by Vaccine (2017)

Age Group	Influenza	Pertussis	HZ	Pneumococcal
	% Vaccinated Each Year[a]	Vaccinated in Last 10 Years[b]	Ever Vaccinated Since 2006[c]	Ever Vaccinated Since 2014[d]
Ages 50-59 years	44.8%	28.0%	0.0%	N/A
Ages 60-64 years	44.8%	28.0%	23.9%	N/A
Ages 65+ years	65.4%	20.4%	37.4%	66.9%

BRFSS, Behavioral Risk Factor Surveillance System; CDC, Centers for Disease Control and Prevention; HZ, herpes zoster; N/A, not applicable; NHIS, National Health Interview Survey; NIS, National Immunization Survey; RZV, recombinant zoster vaccine, adjuvanted; Tdap, tetanus, diphtheria, and acellular pertussis.

[a] CDC;[34] average across 7 seasons (2010/2011-2016/2017) from NIS and BRFSS.

[b] CDC;[35] 2016 NHIS data, in which survey respondents (adults aged 19-64 years and 65+ years) who received tetanus vaccination were asked whether they had received a Tdap vaccine in the last 10 years.

[c] Ages 50-59 years: Assumed that HZ vaccine coverage reaches current coverage level of 60- to 64 year-old age group 5 years after introduction of RZV (i.e., coverage is 0.0% in 2017, 4.8% in 2018 [when RZV is introduced], 9.6% in 2019, 14.3% in 2020, 19.1% in 2021, and 23.9% in 2022). Ages 60-64 years and 65+ years: CDC;[35] 2016 NHIS data, in which survey respondents aged 60-64 years and 65+ years were asked whether they had ever received an HZ vaccine.

[d] Ages 50-59 and 60-64 years: Assumption. Ages 65+ years: CDC;[35] 2016 NHIS data, in which survey respondents aged 65 years and older were asked whether they had ever received a pneumonia vaccine.

Table S6. Vaccine Efficacy and Waning Input Parameter Values by Vaccine

Model Parameter	Base-Case Value	Sources and Notes
**Influenza vaccine**		
Vaccine efficacy (% reduction in influenza cases)	43.9%	CDC;[36] vaccine efficacy for all ages across 8 seasons (2010/2011 to 2017/2018), reflecting seasons when high-dose and adjuvant vaccines have been available
Duration of protection, years	N/A	Assumption, duration of protection is assumed to last 1 season
**Pertussis vaccine**		
Initial efficacy (% reduction in pertussis cases), Year 1	70%	Acosta[20]
Duration of protection, years	5	Kamiya et al.;[37] linear waning is assumed, resulting in an annual 14.0 percentage point decline per year[a]
**Herpes zoster vaccine**		
Vaccine efficacy, ZVL		
Initial efficacy (% reduction in HZ cases), Year 1		Simplified assumptions based on data from ZEST and SPS (Zostavax prescribing information[38])
Ages 60-69 years	63.9%	
Ages 70+ years	30%	
Duration of protection, years		
Ages 60-69 years	12	Morrison et al.;[39] linear waning is assumed, resulting in an annual 5.4 percentage point decline per year
Ages 70+ years	6	
Vaccine efficacy, RZV		
Percentage 2-dose compliant	69.0%	Patterson et al.;[40] based on estimated series completion for Hepatitis B vaccination
Initial efficacy (% reduction in HZ cases), 2-dose, Year 1		ZOE-50 (Lal et al.[41]) and ZOE-70 (Cunningham et al.[42])
Ages 50-69 years	98.4%	
Ages 70+ years	97.8%	
Initial efficacy (% reduction in HZ cases), 1-dose, Year 1		
Ages 50-69 years	90.0%	

Model Parameter	Base-Case Value	Sources and Notes
Ages 70+ years	69.5%	
Initial efficacy (% reduction in HZ cases), weighted average, Year 1		Weighted average between 1-dose and 2-dose efficacy calculated based on estimate of 69.0% second-dose compliance for RZV
Ages 60-69 years	95.8%	
Ages 70+ years	89.1%	
Duration of protection, years		
2-dose		ZOE-50 (Lal et al.[41]) and ZOE-70 (Cunningham et al.[42]), which reported efficacy waning rates for ages 60-69 years of 2.3 percentage points per year after 4 years post vaccination and efficacy waning rates for ages 70+ years of 3.6 percentage points per year[b]
Ages 50-69 years	43	
Ages 70+ years	28	
1-dose		Assumed same annual waning as ZVL (5.4 percentage points per year); a linear waning curve is assumed, resulting in a duration of protection of approximately 13 or 17 years depending on age at vaccination
Ages 50-69 years	17	
Ages 70+ years	13	
Weighted average		Weighted average between 1-dose and 2-dose waning calculated based on estimate of 69.0% second-dose compliance for RZV, resulting in an annual 3.3 percentage point decline per year for individuals aged 50-69 years and an annual 4.2 percentage point decline per year for individuals aged 70+ years
Ages 50-69 years	30	
Ages 70+ years	22	
**Pneumococcal vaccine**		
Vaccine efficacy, PCV-13 + PPSV-23		
Initial efficacy (% reduction in cases [all serotypes]), PCV-13 Year 1		
% reduction in IPD cases	51.8%	Bonten et al.[43] (CAPITA trial, see Table 2)
% reduction in NBPP	24.1%	
Duration of protection, years	15	Assumption, similar to Chen et al.,[44] resulting in an annual 3.5 percentage point decline per year for IPD and an annual 1.6 percentage point decline per year for NBPP cases
Initial efficacy (% reduction in cases [all serotypes]), PPSV-23 Year 1		
% reduction in IPD cases	74%	Moberley et al.[45]
% reduction in NBPP	54%	

Model Parameter	Base-Case Value	Sources and Notes
Duration of protection, years	5	Assumption, resulting in an annual 14.8 percentage point decline per year for IPD and an annual 10.8 percentage point decline per year for NBPP
Combined efficacy	Varies by year	For the first 5 years, the model uses the higher efficacy of either PCV-13 or PPSV-23 (because individuals are assumed to receive both vaccines going forward) with efficacy waning linearly and PPSV-23 waned completely after 5 years. From years 5 to 15, efficacy for PCV-13 (alone) wanes linearly.

CAPITA, Community-Acquired Pneumonia Immunization Trial in Adults; CDC, Centers for Disease Control and Prevention; HZ, herpes zoster; IPD, invasive pneumococcal disease; N/A, not applicable; NBPP, nonbacteremic pneumococcal pneumonia; PCV-13, pneumococcal conjugate vaccine; PPSV-23, pneumococcal polysaccharide vaccine; RZV, recombinant zoster vaccine, adjuvanted; SPS, Shingles Prevention Study; ZEST, Zostavax Efficacy and Safety Trial; ZOE-50, Zoster Efficacy Study in Adults 50 Years of Age or Older; ZOE-70, Zoster Efficacy Study in Adults 70 Years of Age or Older; ZVL, zoster vaccine live.

[a] Resulting in average effectiveness of 47.5% over the first four years following vaccination, which is similar to effectiveness found or assumed in other studies (Klein et al.;[46] Koepke et al.;[47] Acosta et al.[48]).

[b] Cunningham et al.[42] and Lal et al.[41] showed 1.1 percentage point and 2.3 percentage point waning per year for individuals aged less than 70 years, 3.6 percentage point waning per year for individuals aged 70 years and older.

Table S7. Productivity Loss Costs Due to Disease-related Death (2018 USD)[a]

Model Parameter	Base-Case Value	Sources and Notes
Annual (market and non-market) productivity		
Ages 50-54 years	$87,588	Grosse et al.[14,b]
Ages 55-64 years	$71,443	
Ages 65-74 years	$40,460	
Ages 75-89 years	$16,831	
Ages 90+ years	$0	
Productivity loss costs due to death occurring in Year 1		
Discounted		Calculated based on remaining life expectancy at age of death (Arias et al.[13]) and discounted lifetime remaining earnings based on age-specific productivity estimates from Grosse et al.[14,b]
Age 50 years	$1,153,631	
Age 65 years	$417,234	
Age 80 years	$104,729	
Undiscounted		
Age 50 years	$1,557,621	
Age 65 years	$513,358	
Age 80 years	$116,765	

USD, United States dollars.

[a] Lifetime earnings lost due to death were discounted over the modeled time horizon from the year of death. For example, if an individual aged 50 years died in Year 10 of the model, the lifetime earnings of $1,153,631 were discounted to the year of the analysis (Year 1). Discounted lifetime earnings are applied in the discounted burden of disease results, which are presented in Supplemental Table S10.

[b] Productivity amounts in Grosse et al.[14]were presented in 2016 USD. Productivity amounts were inflated to 2018 USD using the employment cost index.[16]

S-3: Model Validation of 1-Year Burden of Disease Results

A 1-year burden of disease analysis was conducted to validate the underlying model structure, inputs, and calculations with other models in the literature. This 1-year burden of disease analysis (i.e., focusing on results from modeled year 2017) was compared to the 1-year analysis by McLaughlin et al.[11]. and Ozawa et al.[49]. This comparison provides context for the projected increase in costs over time due to the aging of the US population.

In Year 1, the model estimated an undiscounted burden of disease that included 7,572,409 cases of influenza, 237,296 cases of pertussis, 994,153 cases of HZ, and 591,419 cases of pneumococcal disease, Supplemental Table 10. The direct medical costs associated with each disease amounted to approximately $6.9 billion for influenza, $267.2 million for pertussis, $2.1 billion for HZ, and $7.2 billion for pneumococcal disease. From the societal perspective, burden of disease cost estimates were substantially higher, with estimated total costs of $14.2 billion for influenza, $497.9 million for pertussis, $6.1 billion for HZ, and $14.0 billion for pneumococcal disease. Across all four diseases in Year 1, the model estimated nearly 26,000 disease-related deaths, with a total disease burden of approximately $16.6 billion from the direct medical cost perspective and $34.9 billion from the societal perspective.

Results from this 1-year burden of disease analysis were within the range of those reported from the previous analyses by McLaughlin et al. and Ozawa et al. The current model estimated approximately 7.6 million cases of influenza, 237,000 cases of pertussis, 994,000 cases of HZ, and 590,000 cases of pneumococcal disease. McLaughlin et al. estimated approximately 8.1 million cases of influenza, 388,000 cases of pertussis, 938,000 cases of HZ, and 603,000 cases of pneumococcal disease. Among the same age group of individuals aged 50 years and older, Ozawa et al. estimated approximately 6.8 million cases of influenza, only 2,151 cases of pertussis, 1.1 million cases of HZ, and 268,000 cases of pneumococcal disease.

Although the current model estimated numbers of cases that fell within the range of those reported by McLaughlin et al. and Ozawa et al., estimates were more similar to those reported by McLaughlin et al. and larger differences were observed as compared with estimated numbers of cases from Ozawa et al. Specifically, the current model estimated approximately 767,000 more cases of influenza, 235,000 more cases of pertussis, and 322,000 more cases of pneumococcal disease as compared with Ozawa et al. For pertussis, this difference is largely explained by a difference in methodology, where the current analysis and McLaughlin et al. estimated the number of cases of pertussis accounting for underreporting; the study by Ozawa et al. limited pertussis cases to those reported by US surveillance data. For influenza and pneumococcal disease, differences in numbers of

cases as compared with Ozawa et al. were due to differences in input parameter values used (e.g., disease incidence, vaccine coverage, vaccine effectiveness).

As compared with previously published estimates from McLaughlin et al. among individuals aged 50 years and older, associated direct medical costs estimated by the current study were lower for influenza (approximately $6.8 billion vs. $12.8 billion) but higher for pertussis ($262.1 million vs. $167.6 million), HZ ($2.1 billion vs. $1.9 billion), and pneumococcal disease ($5.0 billion vs. $4.6 billion). Across all four diseases, the current study estimated lower total direct medical costs than McLaughlin et al.[11,49] among individuals aged 50 years and older (approximately $16.6 billion vs. $19.4 billion). From the societal perspective, 1-year burden of disease cost estimates from the current study were higher than previous estimates for influenza, pertussis, HZ, and pneumococcal disease, with a total disease burden of approximately $34.9 billion versus $26.5 billion reported by McLaughlin et al.

Because the current model estimated numbers of cases similar to those reported by McLaughlin et al., differences in cost estimates are likely due to differences in the assumed severity distribution of cases and/or differences in specific indirect and direct costs included in the analyses. McLaughlin et al. note as a limitation that their model did not take into account indirect costs associated with premature mortality due to disease. These costs were included in the current study, with total costs of productivity loss due to mortality estimated at approximately $9.8 billion across the four diseases. Without these costs, our model estimates total costs of approximately $25.0 billion from the societal perspective, which is slightly lower than the estimate of $26.5 billion from McLaughlin et al. Costs from McLaughlin et al. were also reported in 2013 US dollars, whereas the current analysis estimated costs in 2018 US dollars.

It is difficult to compare direct cost estimates from the current study with the previous analysis by Ozawa et al. [reported in 2011 US dollars] because direct cost estimates for individuals aged 50 years and older were not provided by Ozawa et al. Societal costs were presented for individuals aged 50 years and older and estimates from the current study are substantially higher than those estimated by Ozawa et al. even after excluding societal costs related to disease mortality that were not included in the Ozawa et al. analysis ($25.0 billion vs. $6.6 billion). This large difference is at least partially explained by the differences in estimated number of cases of disease (particularly for pertussis and pneumococcal disease) as discussed above; however, the difference is also due to different references and values used for the direct cost per case to treat each of the four diseases and the indirect cost per case related to illness. For direct costs, Ozawa et al. used the cost of an inpatient case from the

Healthcare Cost and Utilization Project (HCUP) database and a cost per case plus cost of medications from the national Medical Expenditure Panel Survey (MEPS) survey. Parameter values for the current study were more aligned with the approach by McLaughlin et al. that used data from the most recent literature for each disease. In fact, the current study used the same reference and methodology as that used by McLaughlin et al. for both influenza (Molinari et al.[9]) and herpes zoster (Yawn et al.[27]). These direct costs per case were generally higher than those used by Ozawa et al. and were deemed more comprehensive estimates that potentially captured costs related to inpatient cases that may not have been captured in the costing approach used by Ozawa et al. (for example, follow-up outpatient care after hospitalization). Similarly, the indirect costs per case used in the current study were generally better aligned with the higher cost per case used in the McLaughlin et al. study than estimated in the study by Ozawa et al. Both studies applied a standard wage from the US Bureau of Labor statistics, so differences would be explained by different assumptions in the number of days of lost productivity.

Table S8. Burden of Disease Analysis Detailed Results, by Age and Overall: 1-Year Time Horizon

	Age (Years)			
**Disease/Outcomes**	**50-59**	**60-64**	**65+**	**Total**
**Influenza**				
Number of cases				
Hospitalized cases	20,119	9,287	165,206	194,612
ED cases	74,729	34,494	16,858	126,080
Outpatient cases	1,028,957	474,954	1,443,020	2,946,931
NMA cases	1,750,376	807,952	1,746,459	4,304,787
**Total cases**	**2,874,181**	**1,326,687**	**3,371,542**	**7,572,409**
Number of disease-related deaths	642	296	9,697	10,635
Direct medical costs (paid by payer)				
Hospitalized cases	$937,961,289	$432,951,396	$3,817,595,342	$5,188,508,027
ED cases	$27,024,172	$12,474,025	$6,096,260	$45,594,457
Outpatient cases	$574,771,010	$265,307,230	$851,779,395	$1,691,857,635
NMA cases	$0	$0	$0	$0
**Total direct medical costs**	**$1,539,756,471**	**$710,732,652**	**$4,675,470,997**	**$6,925,960,120**
Direct costs (paid by patient) and indirect costs				
OTC medication costs	$8,751,881	$4,039,761	$8,732,293	$21,523,935
Productivity loss due to disease	$2,055,877,517	$948,967,780	$802,547,590	$3,807,392,886
Productivity loss due to mortality	$774,167,101	$210,330,959	$2,521,845,367	$3,506,343,428
**Total direct costs paid by patient and indirect costs**	**$2,838,796,499**	**$1,163,338,499**	**$3,333,125,251**	**$7,335,260,249**
**Total societal costs**	**$4,378,552,970**	**$1,874,071,151**	**$8,008,596,248**	**$14,261,220,369**
**Pertussis**				
Number of cases				
Severe cases	3,123	1,435	10,244	14,802

Disease/Outcomes	Age (Years) 50-59	60-64	65+	Total
Moderate cases	89,527	41,130	63,173	193,830
Mild cases	11,451	5,261	11,952	28,664
NMA cases	0	0	0	0
**Total cases**	**104,101**	**47,826**	**85,369**	**237,296**
Number of disease-related deaths	27	12	88	127
Direct medical costs (paid by payer)				
Severe cases	$44,479,996	$20,435,064	$145,905,247	$210,820,307
Moderate cases	$25,069,786	$11,517,597	$17,690,091	$54,277,475
Mild cases	$849,212	$390,146	$886,335	$2,125,693
NMA cases	$0	$0	$0	$0
**Total direct medical costs**	**$70,398,995**	**$32,342,807**	**$164,481,673**	**$267,223,475**
Direct costs (paid by patient) and indirect costs				
OTC medication costs	$0	$0	$0	$0
Productivity loss due to disease	$73,106,273	$33,586,589	$59,951,606	$166,644,468
Productivity loss due to mortality	$32,402,613	$8,762,040	$22,911,602	$64,076,256
**Total direct costs paid by patient and indirect costs**	**$105,508,887**	**$42,348,629**	**$82,863,208**	**$230,720,724**
**Total societal costs**	**$175,907,881**	**$74,691,436**	**$247,344,881**	**$497,944,199**
**Herpes zoster**				
Number of cases				
Complicated cases, with PHN or nonpain complications	35,505	25,643	128,589	189,737
Uncomplicated cases	257,877	136,195	410,344	804,415
**Total cases**	**293,382**	**161,838**	**538,933**	**994,153**
Number of disease-related deaths	0	0	0	0
Direct medical costs (paid by payer)				

Disease/Outcomes	Age (Years)			
	50-59	60-64	65+	Total
Complicated cases, with PHN or nonpain complications	$124,284,764	$183,706,653	$902,255,427	$1,210,246,844
Uncomplicated cases	$236,085,014	$151,585,819	$547,376,624	$935,047,457
**Total direct medical costs**	**$360,369,778**	**$335,292,472**	**$1,449,632,051**	**$2,145,294,301**
Direct costs (paid by patient) and indirect costs				
OTC medication costs	$0	$0	$0	$0
Productivity loss due to disease	$1,079,148,747	$811,862,094	$2,073,566,963	$3,964,577,804
Productivity loss due to mortality	$0	$0	$0	$0
**Total direct costs paid by patient and indirect costs**	**$1,079,148,747**	**$811,862,094**	**$2,073,566,963**	**$3,964,577,804**
**Total societal costs**	**$1,439,518,525**	**$1,147,154,566**	**$3,523,199,013**	**$6,109,872,104**
**Pneumococcal disease**				
Number of cases				
Meningitis	485	224	764	1,473
Bacteremia	7,089	3,272	11,180	21,542
NBPP - inpatient	33,717	15,563	164,367	213,647
NBPP - outpatient	78,351	36,166	240,239	354,757
**Total cases**	**119,642**	**55,225**	**416,551**	**591,419**
Number of disease-related deaths	1,887	871	12,789	15,546
Direct medical costs (paid by payer)				
Meningitis	$22,195,298	$10,245,076	$23,617,335	$56,057,709
Bacteremia	$324,606,229	$149,834,243	$345,403,517	$819,843,989
NBPP - inpatient	$1,343,467,157	$620,127,918	$4,365,650,820	$6,329,245,895
NBPP - outpatient	$11,344,973	$5,236,700	$69,571,529	$86,153,202
**Total direct medical costs**	**$1,701,613,656**	**$785,443,938**	**$4,804,243,201**	**$7,291,300,795**
Direct costs (paid by patient) and indirect costs				

Disease/Outcomes	Age (Years) 50-59	60-64	65+	Total
OTC medication costs	$0	$0	$0	$0
Productivity loss due to disease	$153,173,981	$70,703,226	$228,418,392	$452,295,598
Productivity loss due to mortality	$2,276,367,701	$618,458,988	$3,367,278,766	$6,262,105,455
**Total direct costs paid by patient and indirect costs**	**$2,429,541,682**	**$689,162,213**	**$3,595,697,158**	**$6,714,401,054**
**Total societal costs**	**$4,131,155,338**	**$1,474,606,151**	**$8,399,940,359**	**$14,005,701,849**
**Total burden of disease**				
Number of disease-related deaths	2,555	1,179	22,574	26,309
Direct medical costs (paid by payer)	$3,672,138,899	$1,863,811,869	$11,093,827,922	$16,629,778,691
Direct costs (paid by patient) and indirect costs	$6,452,995,815	$2,706,711,435	$9,085,252,580	$18,244,959,830
**Total societal costs**	**$10,125,134,714**	**$4,570,523,304**	**$20,179,080,502**	**$34,874,738,521**

ED, emergency department; NMA, nonmedically attended; NBPP, nonbacteremic pneumococcal pneumonia; OTC, over-the-counter; PHN, postherpetic neuralgia; USD, United States dollars.

Note: All cost outcomes are in 2018 USD.

S-4: Detailed 30-Year Burden of Disease Analysis Results

Table S9. Burden of Disease Analysis Detailed Results (Undiscounted), by Age and Overall: 30-Year Time Horizon

	Age (Years)			
**Disease/Outcomes**	**50-59**	**60-64**	**65+**	**Total**
**Influenza**				
Number of cases				
Hospitalized cases	599,533	283,481	7,043,407	7,926,422
ED cases	2,226,837	1,052,931	718,715	3,998,483
Outpatient cases	30,661,831	14,498,048	61,522,005	106,681,884
NMA cases	52,159,371	24,662,881	74,458,875	151,281,127
**Total cases**	**85,647,572**	**40,497,341**	**143,743,003**	**269,887,916**
Number of disease-related deaths	19,122	9,041	413,431	441,594
Direct medical costs (paid by payer)				
Hospitalized cases	$27,950,260,119	$13,215,917,125	$162,760,137,908	$203,926,315,152
ED cases	$805,291,900	$380,771,806	$259,909,195	$1,445,972,901
Outpatient cases	$17,127,571,718	$8,098,549,617	$36,314,936,343	$61,541,057,677
NMA cases	$0	$0	$0	$0
**Total direct medical costs**	**$45,883,123,737**	**$21,695,238,548**	**$199,334,983,446**	**$266,913,345,731**
Direct costs (paid by patient) and indirect costs				
OTC medication costs	$260,796,857	$123,314,403	$372,294,377	$756,405,637
Productivity loss due to disease	$61,262,988,190	$28,967,407,505	$34,215,977,516	$124,446,373,212
Productivity loss due to mortality	$23,232,073,471	$6,394,466,188	$96,314,364,797	$125,940,904,455
**Total direct costs paid by patient and indirect costs**	**$84,755,858,518**	**$35,485,188,096**	**$130,902,636,690**	**$251,143,683,304**
**Total societal costs**	**$130,638,982,255**	**$57,180,426,644**	**$330,237,620,136**	**$518,057,029,035**
**Pertussis**				
Number of cases				

Disease/Outcomes	Age (Years)			
	50-59	60-64	65+	Total
Severe cases	92,981	43,933	437,584	574,498
Moderate cases	2,665,452	1,259,426	2,698,434	6,623,311
Mild cases	340,930	161,089	510,514	1,012,534
NMA cases	0	0	0	0
**Total cases**	**3,099,362**	**1,464,449**	**3,646,532**	**8,210,343**
Number of disease-related deaths	800	378	3,763	4,941
Direct medical costs (paid by payer)				
Severe cases	$1,324,290,643	$625,727,477	$6,232,337,658	$8,182,355,779
Moderate cases	$746,395,820	$352,672,108	$755,631,649	$1,854,699,577
Mild cases	$25,283,357	$11,946,389	$37,859,752	$75,089,499
NMA cases	$0	$0	$0	$0
**Total direct medical costs**	**$2,095,969,820**	**$990,345,974**	**$7,025,829,059**	**$10,112,144,854**
Direct costs (paid by patient) and indirect costs				
OTC medication costs	$0	$0	$0	$0
Productivity loss due to disease	$2,176,572,869	$1,028,430,923	$2,560,830,797	$5,765,834,590
Productivity loss due to mortality	$971,515,968	$267,212,694	$876,585,952	$2,115,314,614
**Total direct costs paid by patient and indirect costs**	**$3,148,088,837**	**$1,295,643,617**	**$3,437,416,750**	**$7,881,149,204**
**Total societal costs**	**$5,244,058,657**	**$2,285,989,591**	**$10,463,245,809**	**$17,993,294,058**
**Herpes zoster**				
Number of cases				
Complicated cases, with PHN or nonpain complications	857,845	761,567	5,259,375	6,878,787
Uncomplicated cases	6,230,662	4,044,765	16,300,101	26,575,529
**Total cases**	**7,088,507**	**4,806,332**	**21,559,476**	**33,454,315**
Number of disease-related deaths	0	0	0	0
Direct medical costs (paid by payer)				

Disease/Outcomes	Age (Years)			
	50-59	60-64	65+	Total
Complicated case, with PHN or nonpain complications	$3,002,886,912	$5,455,801,071	$36,869,291,105	$45,327,979,089
Uncomplicated cases	$5,704,131,191	$4,501,862,409	$22,010,944,109	$32,216,937,710
**Total direct medical costs**	**$8,707,018,103**	**$9,957,663,481**	**$58,880,235,215**	**$77,544,916,799**
Direct costs (paid by patient) and indirect costs				
OTC medication costs	$0	$0	$0	$0
Productivity loss due to disease	$26,073,683,921	$24,111,037,952	$81,122,610,047	$131,307,331,920
Productivity loss due to mortality	$0	$0	$0	$0
**Total direct costs paid by patient and indirect costs**	**$26,073,683,921**	**$24,111,037,952**	**$81,122,610,047**	**$131,307,331,920**
**Total societal costs**	**$34,780,702,024**	**$34,068,701,432**	**$140,002,845,262**	**$208,852,248,718**
**Pneumococcal disease**				
Number of cases				
Meningitis	14,445	6,830	36,365	57,640
Bacteremia	211,252	99,888	531,844	842,983
NBPP - inpatient	1,004,737	475,077	7,626,163	9,105,976
NBPP - outpatient	2,334,787	1,103,974	11,146,442	14,585,203
**Total cases**	**3,565,219**	**1,685,768**	**19,340,814**	**24,591,801**
Number of disease-related deaths	56,226	26,586	595,442	678,253
Direct medical costs (paid by payer)				
Meningitis	$661,396,533	$312,732,752	$1,123,473,656	$2,097,602,941
Bacteremia	$9,672,924,288	$4,573,716,495	$16,430,802,225	$30,677,443,008
NBPP - inpatient	$40,033,908,575	$18,929,513,202	$202,554,028,031	$261,517,449,808
NBPP - outpatient	$338,068,261	$159,851,182	$3,227,925,010	$3,725,844,453
**Total direct medical costs**	**$50,706,297,657**	**$23,975,813,631**	**$223,336,228,923**	**$298,018,340,210**
Direct costs (paid by patient) and indirect costs				
OTC medication costs	$0	$0	$0	$0

Disease/Outcomes	Age (Years) 50-59	60-64	65+	Total
Productivity loss due to disease	$4,564,423,558	$2,158,228,339	$10,150,656,838	$16,873,308,735
Productivity loss due to mortality	$68,311,791,603	$18,802,344,189	$134,929,653,066	$222,043,788,858
**Total direct costs paid by patient and indirect costs**	**$72,876,215,161**	**$20,960,572,528**	**$145,080,309,904**	**$238,917,097,593**
**Total societal costs**	**$123,582,512,818**	**$44,936,386,159**	**$368,416,538,827**	**$536,935,437,804**
**Total burden of disease**				
Number of disease-related deaths	76,147	36,005	1,012,636	1,124,788
Direct medical costs (paid by payer)	$107,392,409,318	$56,619,061,633	$488,577,276,643	$652,588,747,594
Direct costs paid by patient and indirect costs	$186,853,846,436	$81,852,442,193	$360,542,973,391	$629,249,262,020
**Total societal costs**	**$294,246,255,754**	**$138,471,503,826**	**$849,120,250,034**	**$1,281,838,009,614**

ED, emergency department; NBPP, nonbacteremic pneumococcal pneumonia; NMA, nonmedically attended; OTC, over-the-counter; PHN, postherpetic neuralgia; USD, United States dollars.

Note: All cost outcomes are in 2018 USD and are presented undiscounted.

Table S10. Burden of Disease Costs (Discounted): 30-Year Time Horizon

Disease/Outcomes	Discounted Costs
**Influenza**	
Total direct medical costs (paid by payer)	$174,400,963,034
Total direct costs paid by patient and indirect costs	$152,234,704,696
Total societal costs	$326,635,667,730
**Pertussis**	
Total direct medical costs	$6,616,969,154
Total direct costs paid by patient and indirect costs	$4,932,824,691
Total societal costs	$11,549,793,845
**Herpes zoster**	
Total direct medical costs	$50,606,887,823
Total direct costs paid by patient and indirect costs	$86,538,056,497
Total societal costs	$137,144,944,320
**Pneumococcal disease**	
Total direct medical costs	$193,821,284,462
Total direct costs paid by patient and indirect costs	$131,434,038,716
Total societal costs	$325,255,323,178
**Total burden of disease**	
Direct medical costs (paid by payer)	$425,446,104,473
Direct costs paid by patient and indirect costs	$375,139,624,599
Total societal costs	$800,585,729,072

References

1. Kostova D, Reed C, Finelli L, Cheng P-Y, Gargiullo PM, Shay DK, Singleton JA, Meltzer MI, Lu P-J, Bresee JS. Influenza Illness and Hospitalizations Averted by Influenza Vaccination in the United States, 2005-2011. PLoS One 2013; 8:e66312-e66312.
2. Bresee J, Reed C, Kim IK, Finelli L, Fry A, Chaves SS, Burns E, Gargiullo P, Jernigan D, Cox N, et al. Estimated Influenza Illnesses and Hospitalizations Averted by Influenza Vaccination — United States, 2012–13 Influenza Season. MMWR Morb Mortal Wkly Rep 2013; 62:997-1000.
3. Reed C, Kim IK, Singleton JA, Chaves SS, Flannery B, Finelli L, Fry A, Burns E, Gargiullo P, Jernigan D, et al. Estimated influenza illnesses and hospitalizations averted by vaccination — United States, 2013–14 influenza season. MMWR Morb Mortal Wkly Rep 2014; 63:1151-1154.
4. Centers for Disease Control and Prevention. Estimated Influenza Illnesses and Hospitalizations Averted by Vaccination — United States, 2014–15 Influenza Season. 2015. [accessed: 2016 Apr 20]. http://www.cdc.gov/flu/about/disease/2014-15.htm.
5. Centers for Disease Control and Prevention. Estimated Influenza Illnesses, Medical Visits, Hospitalizations, and Deaths Averted by Vaccination in the United States. 2017. [accessed: 2019 Mar 28]. https://www.cdc.gov/flu/about/disease/2015-16.htm.
6. Project HCaU. Rockville, MD: Agency for Healthcare Research and Quality. 2019. [accessed: 2019 Dec 6]. https://hcupnet.ahrq.gov/.
7. Talbird SE, La EM, Poston SA, Hogea CS. A budget-impact analysis of quadrivalent influenza vaccine use in the United States. Value Health 2017; 20:A73.
8. Reed C, Chaves SS, Daily Kirley P, Emerson R, Aragon D, Hancock EB, Butler L, Baumbach J, Hollick G, Bennett NM, et al. Estimating influenza disease burden from population-based surveillance data in the United States. PLoS One 2015; 10:e0118369-e0118369.
9. Molinari N-AM, Ortega-Sanchez IR, Messonnier ML, Thompson WW, Wortley PM, Weintraub E, Bridges CB. The annual impact of seasonal influenza in the US: Measuring disease burden and costs. Vaccine 2007; 25:5086-5096.
10. Karve S, Meier G, Davis KL, Misurski DA, Wang C-C. Influenza-related health care utilization and productivity losses during seasons with and without a match between the seasonal and vaccine virus B lineage. Vaccine 2013; 31:3370-3388.
11. McLaughlin JM, McGinnis JJ, Tan L, Mercatante A, Fortuna J. Estimated Human and Economic Burden of Four Major Adult Vaccine-Preventable Diseases in the United States, 2013. J Prim Prev 2015; 36:259-273.
12. Grosse SD, Krueger KV, Mvundura M. Economic Productivity by Age and Sex: 2007 Estimates for the United States. Medical Care 2009; 47:S94-S103.
13. Arias E, Heron M, Xu JQ. United States life tables, 2015. Hyattsville, MD: National Center for Health Statistics, 2018.
14. Grosse SD, Krueger KV, Pike J. Estimated annual and lifetime labor productivity in the United States, 2016: implications for economic evaluations. J Med Econ 2019; 22:501-508.
15. Statistics UBoL. Consumer Price Index for All Urban Consumers: Medical Care [CUSR0000SAM]. 2019. [accessed: 2019 Sep 20]. https://www.bls.gov/cpi/data.htm.

16. Statistics UBoL. Employment Cost Index - Total compensation for All Civilian workers in All industries and occupations, Index, 2001-2019. 2019. [accessed: 2019 Aug 22]. https://www.bls.gov/cpi/data.htm.
17. Adams DA, Thomas KR, Jajosky RA, Foster L, Baroi G, Sharp P, Onweh DH, Schley AW, Anderson WJ, for the Nationally Notifiable Infectious Conditions Group. Summary of Notifiable Infectious Diseases and Conditions — United States, 2015. MMWR Morb Mortal Wkly Rep 2017; 64:1-143.
18. Chen C-C, Balderston McGuiness C, Krishnarajah G, Blanchette CM, Wang Y, Sun K, Buck PO. Estimated incidence of pertussis in people aged <50 years in the United States. Hum Vaccin Immunother 2016; 12:2536-2545.
19. Masseria C, Krishnarajah G. The estimated incidence of pertussis in people aged 50 years old in the United States, 2006-2010. BMC Infect Dis 2015; 15:534-534.
20. Acosta A. Cost-effectiveness of pertussis vaccine substitution for tetanus booster in prevention of pertussis in adults 65 years and older. Presentation to the Advisory Committee on Immunization Practices (ACIP) Atlanta, GA: US Department of Health and Human Services, CDC, 2012.
21. Millier A, Aballea S, Annemans L, Toumi M, Quilici S. A critical literature review of health economic evaluations in pertussis booster vaccination. Expert Rev Pharmacoecon Outcomes Res 2012; 12:71-94.
22. Cortese MM, Baughman AL, Brown K, Srivastava P. A "New Age" in Pertussis Prevention: New Opportunities Through Adult Vaccination. American Journal of Preventive Medicine 2007; 32:177-185.e171.
23. Talbird SE, Graham J, Mauskopf J, Masseria C, Krishnarajah G. Impact of Tetanus, Diphtheria, and Acellular Pertussis (Tdap) Vaccine Use in Wound Management on Health Care Costs and Pertussis Cases. Journal of Managed Care & Specialty Pharmacy 2015; 21:88-99.
24. Caro JJ, Getsios D, El-Hadi W, Payne K, O'Brien JA. Pertussis Immunization of Adolescents in the United States: An Economic Evaluation. The Pediatric Infectious Disease Journal 2005; 24:S75-S82.
25. Centers for Medicare & Medicaid Services Physician fee schedule search. CPT 99213, 90471; Data Year: 2018. 2018. [accessed: 2018 Feb 20]. www.cms.gov/apps/physician-fee-schedule/search/search-results.aspx?Y=0&T=0&HT=0&CT=3&H1=90471&M=5.
26. Johnson BH, Palmer L, Gatwood J, Lenhart G, Kawai K, Acosta CJ. Annual incidence rates of herpes zoster among an immunocompetent population in the United States. BMC Infect Dis 2015; 15:502-502.
27. Yawn BP, Itzler RF, Wollan PC, Pellissier JM, Sy LS, Saddier P. Health care utilization and cost burden of herpes zoster in a community population. Mayo Clin Proc 2009; 84:787-794.
28. Centers for Disease Control and Prevention. Active Bacterial Core Surveillance report, Emerging Infections Program Network, Streptococcus pneumoniae, 2013. Atlanta, GA: Centers for Disease Control and Prevention, 2013.
29. Stoecker C, Kim L, Gierke R, Pilishvili T. Incremental Cost-Effectiveness of 13-valent Pneumococcal Conjugate Vaccine for Adults Age 50 Years and Older in the United States. J Gen Intern Med 2016; 31:901-908.
30. Simonsen L, Taylor RJ, Schuck-Paim C, Lustig R, Haber M, Klugman KP. Effect of 13-valent pneumococcal conjugate vaccine on admissions to hospital 2 years after its introduction in the USA: a time series analysis. The Lancet Respiratory Medicine 2014; 2:387-394.

31. Nelson JC, Jackson M, Yu O, Whitney CG, Bounds L, Bittner R, Zavitkovsky A, Jackson LA. Impact of the introduction of pneumococcal conjugate vaccine on rates of community acquired pneumonia in children and adults. Vaccine 2008; 26:4947-4954.
32. Huang SS, Johnson KM, Ray GT, Wroe P, Lieu TA, Moore MR, Zell ER, Linder JA, Grijalva CG, Metlay JP, et al. Healthcare utilization and cost of pneumococcal disease in the United States. Vaccine 2011; 29:3398-3412.
33. Weycker D, Strutton D, Edelsberg J, Sato R, Jackson LA. Clinical and economic burden of pneumococcal disease in older US adults. Vaccine 2010; 28:4955-4960.
34. Centers for Disease Control and Prevention. Influenza vaccination coverage estimates for persons 6 months and older by State, HHS Region, and the United States, National Immunization Survey-Flu (NIS-Flu) and Behavioral Risk Factor Surveillance System (BRFSS), 2010-11 through 2016-17 influenza seasons. 2017. [accessed: 2018 Feb 20]. https://www.cdc.gov/flu/fluvaxview/reportshtml/trends/index.html.
35. Centers for Disease Control and Prevention. AdultVaxView, Vaccination Coverage Among Adults in the United States, National Health Interview Survey, 2016. 2018. [accessed: 2018 Feb 20]. https://www.cdc.gov/vaccines/imz-managers/coverage/adultvaxview/NHIS-2016.html.
36. Centers for Disease Control and Prevention. Past Seasons Vaccine Effectiveness Estimates. 2018. [accessed: 2019 Dec 12]. https://www.cdc.gov/flu/vaccines-work/past-seasons-estimates.html.
37. Kamiya H, Cho B-H, Messonnier ML, Clark TA, Liang JL. Impact and cost-effectiveness of a second tetanus toxoid, reduced diphtheria toxoid, and acellular pertussis (Tdap) vaccine dose to prevent pertussis in the United States. Vaccine 2016; 34:1832-1838.
38. Zostavax prescribing information. [accessed: 2019 Jan 15]. https://www.merck.com/product/usa/pi_circulars/z/zostavax/zostavax_pi2.pdf.
39. Morrison VA, Johnson GR, Schmader KE, Levin MJ, Zhang JH, Looney DJ, Betts R, Gelb L, Guatelli JC, Harbecke R, et al. Long-term persistence of zoster vaccine efficacy. Clinical infectious diseases : an official publication of the Infectious Diseases Society of America 2015; 60:900-909.
40. Patterson BJ, Cheng WY, Trofa AF, Duchesneau A, Macheca M, Masseria C, Duh MS. A Claims-Based Analysis of Hepatitis A, B and A/B Vaccination Series completion and Compliance among US Adults. ISPOR 22nd Annual International Meeting. Boston, MA, USA, 2017.
41. Lal H, Cunningham AL, Godeaux O, Chlibek R, Diez-Domingo J, Hwang S-J, Levin MJ, McElhaney JE, Poder A, Puig-Barberà J, et al. Efficacy of an Adjuvanted Herpes Zoster Subunit Vaccine in Older Adults. 2015; 372:2087-2096.
42. Cunningham AL, Lal H, Kovac M, Chlibek R, Hwang S-J, Díez-Domingo J, Godeaux O, Levin MJ, McElhaney JE, Puig-Barberà J, et al. Efficacy of the Herpes Zoster Subunit Vaccine in Adults 70 Years of Age or Older. N Engl J Med 2016; 375:1019-1032.
43. Bonten MJM, Huijts SM, Bolkenbaas M, Webber C, Patterson S, Gault S, van Werkhoven CH, van Deursen AMM, Sanders EAM, Verheij TJM, et al. Polysaccharide Conjugate Vaccine against Pneumococcal Pneumonia in Adults. N Engl J Med 2015; 372:1114-1125.
44. Chen J, O'Brien MA, Yang HK, Grabenstein JD, Dasbach EJ. Cost-effectiveness of pneumococcal vaccines for adults in the United States. Adv Ther 2014; 31:392-409.
45. Moberley S, Holden J, Tatham DP, Andrews RM. Vaccines for preventing pneumococcal infection in adults. Cochrane Database Syst Rev 2013.
46. Klein NP, Bartlett J, Fireman B, Baxter R. Waning Tdap Effectiveness in Adolescents. Pediatrics 2016; 137:e20153326.

47. Koepke R, Eickhoff JC, Ayele RA, Petit AB, Schauer SL, Hopfensperger DJ, Conway JH, Davis JP. Estimating the Effectiveness of Tetanus-Diphtheria-Acellular Pertussis Vaccine (Tdap) for Preventing Pertussis: Evidence of Rapidly Waning Immunity and Difference in Effectiveness by Tdap Brand. J Infect Dis 2014; 210:942-953.
48. Acosta AM, DeBolt C, Tasslimi A, Lewis M, Stewart LK, Misegades LK, Messonnier NE, Clark TA, Martin SW, Patel M. Tdap vaccine effectiveness in adolescents during the 2012 Washington State pertussis epidemic. Pediatrics 2015; 135:981-989.
49. Ozawa S, Portnoy A, Getaneh H, Clark S, Knoll M, Bishai D, Yang HK, Patwardhan PD. Modeling The Economic Burden Of Adult Vaccine-Preventable Diseases In The United States. Health Aff (Millwood) 2016; 35:2124-2132.
